# Whole genome sequencing and functional annotation of *Fusarium oxysporum* f. sp. *lentis* to unravel virulence and secondary metabolite biosynthesis gene clusters

**DOI:** 10.3389/fgene.2025.1585510

**Published:** 2025-06-18

**Authors:** Sanjay Kharte, Ashish Kumar, Priyamvada Mishra, R. S. Ramakrishnan, Stuti Sharma, Nishi Mishra, Puneet Singh Chauhan, Radheshyam Sharma, Vedant Gautam, Shweta Tiwari, Vinod Goyal, Sonu Sharma, G. K. Koutu, N. K. Joshi

**Affiliations:** ^1^ Department of Plant Pathology, Jawaharlal Nehru Agricultural University, Jabalpur, Madhya Pradesh, India; ^2^ CSIR-National Botanical Research Institute, Lucknow, Uttar Pradesh, India; ^3^ Department of Plant Physiology, Jawaharlal Nehru Agricultural University, Jabalpur, Madhya Pradesh, India; ^4^ Department of Genetics and Plant Breeding, Jawaharlal Nehru Agricultural University, Jabalpur, Madhya Pradesh, India; ^5^ Seed Technology Research Centre, Jawaharlal Nehru Agricultural University, Jabalpur, Madhya Pradesh, India; ^6^ Biotechnology Centre, Jawaharlal Nehru Agricultural University, Jabalpur, Madhya Pradesh, India; ^7^ Department of Entomology and Plant Pathology, University of Arkansas, Fayetteville, AR, United States

**Keywords:** *Fusarium oxysporum* f. sp. *lentis*, illumina shotgun sequencing, pathogenicity, CAZymes, secondary metabolites, virulence factors, host-pathogen interactions, gene annotation

## Abstract

**Background:**

*Fusarium oxysporum* f. sp. *lentis* is a major fungal pathogen that causes vascular wilt in lentil crops, leading to significant reductions in yield. Despite its importance, the genetic underpinnings of this pathogen remain poorly understood.

**Methods:**

We performed whole-genome sequencing of *F. oxysporum* f. sp. *lentis* using the Illumina Shotgun Sequencing platform. The resulting high-quality genome assembly consisted of 12,366 contigs with a total length of 124.48 Mb. Genome completeness was evaluated using Benchmarking Universal Single-Copy Orthologs (BUSCO) analysis, and functional annotation was performed through comparisons with several public databases, including Uniprot, Gene Ontology (GO), Kyoto Encyclopedia of Genes and Genomes (KEGG), Pfam, and Clusters of Orthologous Groups (COG). Pathogenicity-related genes were identified using the PHI-base database, and secondary metabolite biosynthesis was analyzed with AntiSMASH.

**Results:**

The genome assembly achieved 99% completeness, identifying 116,998 protein-coding genes. A total of 16,779 carbohydrate-active enzymes (CAZymes) could be detected, highlighting the pathogen’s potential for plant cell wall degradation. Pathogenicity analysis revealed genes linked with moderate virulence. AntiSMASH detected 77 biosynthetic gene clusters (BGCs), including those encoding Type I polyketide synthases (T1PKS) and non-ribosomal peptide synthetases (NRPS), which may contribute to pathogenicity.

**Discussion:**

The comprehensive genomic analysis of *F. oxysporum* f. sp. *lentis* offers valuable insights into its pathogenic mechanisms, including plant cell wall degradation and secondary metabolite production. These findings pave the way for future research on host-pathogen interactions and the development of targeted disease management strategies.

## 1 Introduction

Lentil (*Lens culinaris* (L.) Medik.) is a nutritionally significant pulse crop cultivated across various regions, including the Indian subcontinent, West Asia, Ethiopia, and North Africa. In India, chickpea, pigeon pea, and lentil are the predominant pulse crops, collectively accounting for over 60% of the nation’s total pulse production. Globally, lentil production reached approximately 5.73 million tons (FAO, 2021). Lentils are an annual, self-pollinating diploid crop characterized by a genome size of approximately 4 Gb (2n = 14) (Arumuganathan and Earle, 1991). Lentil growers worldwide face similar biotic and abiotic stresses which significantly reduce productivity, particularly due to soil and seed-borne diseases, that cause high mortality (Bayaa and Erskine, 1998; Infantino et al., 2006). Notably, strains within the *Fusarium* genus impose substantial constraints on global grain and forage legume production (Rubiales et al., 2015). *Fusarium oxysporum* is a widely distributed soilborne fungal pathogen responsible for vascular wilt diseases in numerous plant species. Its management remains challenging, despite attempts through chemicals (Gordon, 2017; Kharte et al., 2022) and biological control (Garkoti et al., 2013). Among the various biotic stresses affecting lentil yield, wilt disease, caused by *F. oxysporum* f. sp. *lentis,* presents a significant challenge. Addressing this issue is crucial for enhancing lentil production and ensuring higher yields (Choudhary and Mohanka, 2012). In India, wilt is a major concern in the states of Uttar Pradesh, Madhya Pradesh, Bihar, West Bengal, and other lentil growing regions, with reported infections ranging from 25% to 95% (Khare, 1980). Despite its agricultural significance, the molecular basis of pathogenicity, host specificity, and adaptation mechanisms in *F. oxysporum* f. sp. *lentis* remains largely unexplored. Resistant cultivars remain a key strategy for managing lentil wilt, with resistant germplasm serving as valuable input for molecular breeding programs (Kumar et al., 2021).

Several molecular markers have been employed for the characterization and classification of *Fusarium* species (Baayen et al., 1997; Lievens et al., 2008; Baysal et al., 2010; Sharma et al., 2014; van Dam et al., 2016). Additionally, genes associated with virulence have been targeted for the molecular discrimination of fungal strains. (Lievens et al., 2008; van Dam et al., 2016). Many studies have reported that *secreted-in xylem* (SIX) genes, primarily located on a single chromosome, are linked to the pathogenicity of *F. oxysporum* (Rep et al., 2004; Houterman et al., 2007; Kashiwa et al., 2013; Carvalhais et al., 2019). Strains of *F. oxysporum* exhibit variations in gene content, sequences, and chromosome numbers due to rearrangements and the mobility of lineage-specific chromosomes (Davière et al., 2001; Ma et al., 2010; Schmidt et al., 2013; Vlaardingerbroek et al., 2016; Nelson et al., 1983). Analysis of publicly available *F. oxysporum* genome assemblies reveal that genome sizes ranges from approximately 50–70 Mb, varying among various formats. (data of the NCBI Genome database, https://www.ncbi.nlm.nih.gov/genome/browse/#!/eukaryotes/707/). The genome of *F. oxysporum* exhibits high plasticity, comprising a conserved core genome essential for survival and an accessory genome enriched with lineage-specific virulence factors (Ma et al., 2010). The accessory genome contains key pathogenicity-related elements, including SIX effectors, transposable elements, and secondary metabolite gene clusters, all of which contribute to host specificity and adaptation (van Dam et al., 2016). Comparative genomic analyses across different *F. oxysporum* formae speciales have revealed substantial variation in effector repertoires, highlighting the role of horizontal gene transfer in evolution of pathogenicity (Schmidt et al., 2013). Advances in high-throughput sequencing technologies have significantly expanded fungal genomics, enabling a deeper understanding of pathogen diversity, virulence mechanisms, and intricate host-pathogen interactions at the genomic level (Kersey, 2019). Whole-genome sequencing (WGS) offers valuable insights into genetic variations, virulence-associated genes, and the biosynthetic pathways of secondary metabolites that enhance fungal pathogenicity (Schardl et al., 2013). Among sequencing platforms, Illumina sequencing technology has gained widespread acceptance due to its high accuracy and cost-effectiveness, making it suitable for fungal genome sequencing. This technology significantly facilitates *de novo* genome assembly and comparative genomic studies, thereby advancing our understanding of fungal biology (Goodwin et al., 2016). In this study, we employed Illumina-based whole-genome sequencing to generate a high-quality draft genome assembly of *F. oxysporum* f. sp. *lentis*. Genome annotation was performed to identify candidate pathogenicity gene clusters, carbohydrate-active enzymes (CAZymes), and secondary metabolite biosynthetic gene clusters associated with lentil infection. Our findings provide a genomic framework that elucidates the molecular mechanisms underlying *F. oxysporum* pathogenicity and contribute to the development of genome-informed strategies for disease resistance in lentil breeding programs.

## 2 Materials and methods

### 2.1 Fungal strain culture and isolation

The *F. oxysporum* f. sp. *lentis* isolate used in this study was collected from the infected lentil plant showing typical Fusarium wilt symptoms from the wilt infected fields of Damoh, Madhya Pradesh, India. The isolate was cultured on potato dextrose agar (PDA) and incubated at 27°C ± 2°C for 7 days. Fungal mycelia were picked from liquid potato dextrose broth (PDB) cultures grown under shaking conditions (150 rpm) at 25°C for 5 days (Sicard et al., 1997; Nelson et al., 1983).

### 2.2 DNA extraction and purification

The high-quality DNA was extracted from a seven-day-old using the cetyltrimethylammonium bromide (CTAB) method with slight modifications (Doyle and Doyle, 1987). Briefly, mycelia were collected by vacuum filtration, and ground to a fine powder in liquid nitrogen using a mortar and pestle. The fine powdered samples were incubated in DNA extraction buffer (2% CTAB, 1.4 M NaCl, 100 mM Tris-HCl pH 8.0, 20 mM EDTA, and 0.2% β-mercaptoethanol) at 65°C for 45 min. DNA was purified with phenol:chloroform: isoamyl alcohol (25:24:1) solution and precipitated with cold isopropanol. The DNA pellet was washed with 70% ethanol, air-dried, and resuspended in 1X TE buffer. The quantification of extracted DNA was calculated using a Nanodrop spectrophotometer (Thermo Fisher Scientific, United States) and a Qubit fluorometer (Invitrogen, United States). DNA integrity was evaluated by agarose gel electrophoresis (0.8% w/v). Only high-quality DNA (A260/A280 ratio ∼1.8–2.0, and no visible degradation) was preferred for sequencing.

### 2.3 DNA library preparation and sequencing

A single isolate of *Fusarium* was used for Whole Genome Sequencing by Illumina (Illumina Novaseq with 150 × 2 chemistry). The sequencing library was constructed using isolated DNA according to the manufacturer’s instructions in NEBNext^®^ Ultra™ II FS DNA Library Prep Kit for Illumina (https://international.neb.com/protocols/2017/10/25/protocol-for-fs-dna-library-prep-kit-e7805-e6177-with-inputs-greater-than-or-equal--to-100-ng). The quality control of prepared library was executed using Agilent tape station. Subsequently sequenced 150 bp reads at each end using Illumina.

### 2.4 Genome assembly and quality assessment

With 121 k-mer lengths, the Spades assembler version 3.15.5 (Bankevich et al., 2012) performed the *de novo* genome assembly of produced sequence data. The outcomes for reliable assembly were gained after eliminating contigs whose length was less than 200 bp. Using Illumina reads, the complete genome sequence was assembled and then Quake (Kelley et al., 2010) was used for error correction before scaffolding with the help of SOAPdenovo v2.04 (Luo et al., 2012). GapCloser v1.12 (Luo et al., 2012) was laboring to close gaps within the scaffolds. The quality and the completeness of the assembled genome were evaluated through the use of BUSCO v5.3.2 (Benchmarking Universal Single-Copy Orthologs) with the Ascomycota_odb10 database (Simão et al., 2015).

### 2.5 Genome prediction and annotation

Protein coding sequences were predicted via the automated pipeline MAKER2 (v2.31.9) (Holt and Yandell, 2011). The first reiteration of MAKER2 combined evidence from known mRNAs, proteins, and *ab initio* predictions with SNAP (Korf, 2004) and GeneMark-ES v2.3a (Cantarel et al., 2008; Ter-Hovhannisyan et al., 2008). The SNAP hidden Markov models (HMM) were adjusted using the Core Eukaryotic Genes Mapping Approach (CEGMA) output, while GeneMark-ES parameters were self-trained using genome scaffolds >90 bp. The result was then used to train the evidence-based predictor, Augustus (Stanke and Morgenstern, 2005). The resultant gene sets were combined to acquire the most inclusive set of non-redundant reference genes.

### 2.6 Functional annotation

Functional annotation of protein-coding genes was executed based on sequence homology searches counter to major biological databases. The eggNOG-mapper tool (Huerta-Cepas et al., 2019) was utilized to assign Gene Ontology (GO) terms and Kyoto Encyclopedia of Genes and Genomes (KEGG) pathways (https://www.genome.jp/kegg/). In addition, protein models were aligned using BLASTP (Altschul et al., 1997) with an e-value threshold of ≤1e-5, Non-Redundant (NR) database, protein families (Pfam), and SwissProt (BLASTP cut-off e-value≤1e-5), and then classified according to Clusters of Orthologous Groups of proteins (COG), Gene Ontology (GO), and Kyoto Encyclopedia of Genes and Genomes (KEGG) (Kanehisa and Goto, 2000). KOBAS software was used to test the statistical enrichment of differentially expressed genes in KEGG pathways (Xie et al., 2011). The Gene Ontology (GO) terms were used for tree hierarchical classification. KEGG pathway analysis assisted metabolic functions, and PHI-base (Pathogen-Host Interaction Database) (https://www.phi-base.org/) was used to identify putative pathogenicity-related genes (Urban et al., 2020). Repetitive sequences were soft-masked using the Tantan tool integrated into the Funannotate pipeline to retain genome integrity during gene prediction (Palmer and Stajich, 2017). Initially, raw genomic sequences were preprocessed using the funannotate clean command to remove contaminant or low-quality sequences. Tantan was then employed to identify and soft-mask simple repeats by converting them to lowercase letters, thereby preserving sequence length and positional information without altering the underlying sequence data. The masked genome was subsequently used as input for funannotate predict, ensuring accurate gene annotation while accounting for repetitive regions. Further, tRNA genes were predicted using *tRNAscan-SE*, integrated within the Funannotate pipeline, to support comprehensive genome annotation. The analysis began with genome preparation, followed by execution of the funannotate predict module, which incorporates *tRNAscan-SE* for the identification of tRNA genes. The resulting annotations were reviewed to confirm the presence and classification of tRNA loci, contributing to the overall gene annotation profile of the genome.

### 2.7 Identification of carbohydrate-active enzymes

Carbohydrate-active enzymes (CAZymes) in the *F. oxysporum* f. sp. *lentis* genome were recognized using the CAZy database (Lombard et al., 2014). Protein sequences were interrogated against the CAZy database using BLASTP with DIAMOND and HMMscan, applying an e-value threshold of e-value ≤1e−5. Predicted CAZymes were classified into glycoside hydrolases (GHs), glycosyltransferases (GTs), carbohydrate-binding modules (CBMs), polysaccharide lyases (PLs), carbohydrate esterases (CEs), and auxiliary activities (AAs) based on their catalytic functions.

### 2.8 Identification of secondary metabolite gene clusters

Biosynthetic gene clusters (BGCs) related to secondary metabolite (SM) production in *F. oxysporum* f. sp. *lentis* were recognized using the fungal antiSMASH v7.0 beta pipeline (Blin et al., 2023). Default parameters were selected for the analysis. AntiSMASH employs profile hidden Markov models (HMMs) to precisely detect all known secondary metabolite gene clusters.

To further illustrate the identified gene clusters, BLASTP searches were executed against the NCBI Genome Portal Software Platform (NCBI, 2023), and functional annotations were assigned. The known gene clusters were classified into polyketide synthases (PKSs), non-ribosomal peptide synthetases (NRPSs), hybrid PKS-NRPSs, terpene synthases, and other known biosynthetic gene clusters.

### 2.9 Synteny Analysis

Whole-genome protein sequences and gene positions for *F. oxysporum* f. sp. *coagulans* (reference strain) were retrieved from the NCBI Genome Database [Accession number: GCA_014154955.1, GCA_014154955.1_SMRT_HiC_Fo5176_genomic] (NCBI, 2023). If a gene had numerous transcripts, only the first transcript was taken for analysis. Comparative genomic assessment was examined to evaluate gene synteny between *F. oxysporum* F. sp. *lentis* and the reference genome. Protein-coding genes were compared using BLASTP, retentive the top five non-self-hits per target genome with an e-value threshold of e-value≤1e−5. Synteny analysis was performed by MCScanX package (Wang et al., 2012), aiding the documentation of conserved gene blocks and genome rearrangements.

## 3 Result

**FIGURE 1:**
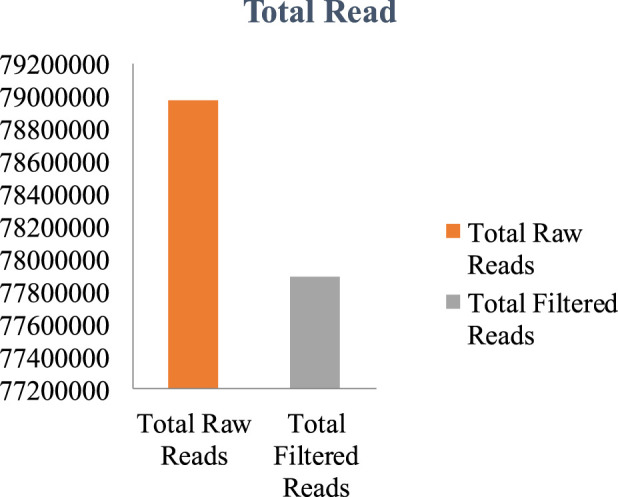
Whole genome assembly- Total raw and filtered reads.

**FIGURE 2:**
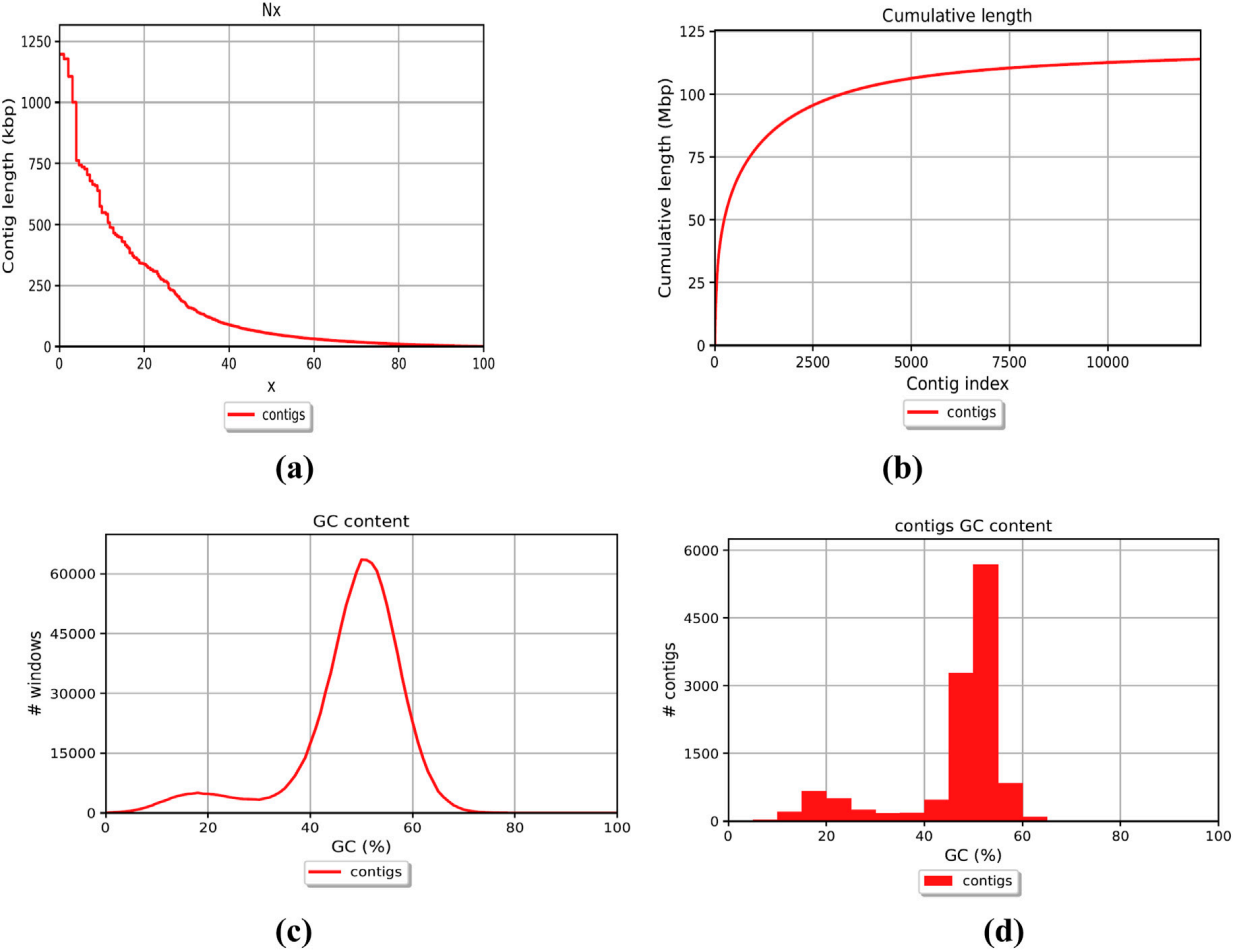
Whole genome assembly; Contigs representation **(a)** Contig length **(b)** Contig’s cumulative length **(c)** Contig GC percent **(d)** Contig’s coverage histogram.

The genome of *F. oxysporum* f. sp. *lentis* was sequenced using a whole-genome shotgun strategy on the Illumina platform. A total of 157,942,234 reads (Figure 1) were generated, comprising 78,971,117 paired-end reads (150 bp each). Subsequently quality control and preprocessing, 77,890,107 clean reads (ranging from 36 to 150 bp) were reserved, with a GC content of 48% in raw reads and 47% in clean reads (Table: 1). *De novo* assembly of the processed reads resulted in 12,366 contigs. The majority of contigs (65,981) ranged from 1 to 1,000 bp in length, followed by 7,992 contigs in the 1,001–5,000 bp range (Figures 2A–D). The total assembly spanned 114,006,452 bp, with the largest contig measuring 1,197,421 bp. The highest total contig length (124,483,966 bp) was observed in the 1–1,000 bp range. The assembly yielded an N50 value of 51,992 bp and an L50 of 353 (Table 2), indicating a high-quality genome assembly.

**TABLE 1:** Quality control attributes of reads of sequence generated.

Sample	Quality control			Trimmed sequences		
	Raw reads	GC %	Read length	Clean reads	GC %	Read length
*Fusarium* sp	78,971,117	48	150	77,890,107	47	36–150

**TABLE 2:** Whole genome sequencing assembly statistics.

Feature	Number of contigs	Total length (bases)
1–1,000 bp	65,981	124,483,966
1,001–5,000 bp	7,992	111,018,751
5,001–10000 bp	3,273	100,319,835
10,001–25000 bp	1,929	90,773,892
25,001–50000 bp	804	73,053,112
>50,000 bp	369	57,866,741
Total number of contigs defining full length in assembly		12,366
Largest contig		1,197,421
Total length		114,006,452
GC %		47.95
N50		51,992
N75		13,766
L50		353
L75		1,479

### 3.1 Gene prediction and annotation

**FIGURE 3:**
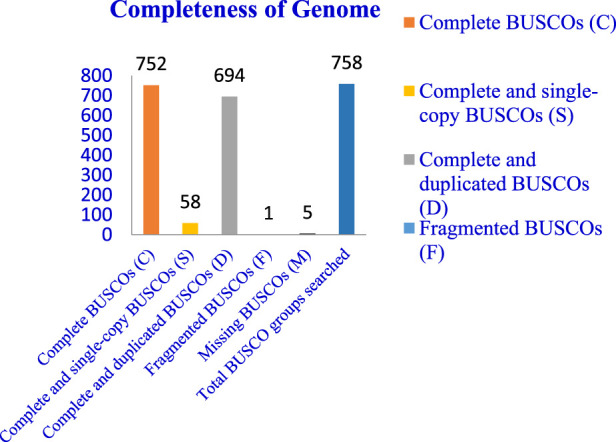
Whole genome assembly analysis: Completeness of genome–BUSCO (Benchmarking Universal Single-Copy Orthologs) analysis.

**FIGURE 4:**
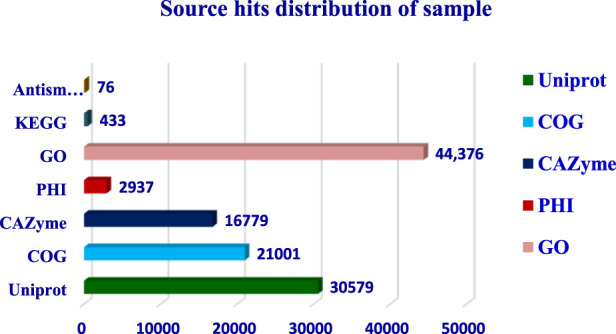
Whole genome assembly analysis: Gene prediction and Functional annotation.

The completeness of the assembled genome was estimated via Benchmarking Universal Single-Copy Orthologs (BUSCO) analysis, which specified a 99% completeness score. Of the 758 BUSCO groups analyzed, 58 were recognized as complete single-copy BUSCOs, 694 as complete duplicated BUSCOs, 1 as a fragmented BUSCO, and 5 as missing BUSCOs (Figure 3; Table 3). A total of 116,998 protein-coding genes were predicted in the *F. oxysporum* genome. Functional annotation of the predicted genes identified homologies with known databases such as UniProt; 30,579 genes (26.14%), Gene Ontology (GO); 44,376 genes (37.93%), KEGG Pathways; 433 genes (0.37%), Pfam (Protein Families Database); 209,329 genes, Clusters of Orthologous Groups (COG); 21,001 genes (17.95%), Carbohydrate-Active Enzymes (CAZymes); 16,779 genes (14.34%), PHI-base (Pathogen-Host Interactions Database); 2,937 genes (2.51%), and antiSMASH (Secondary Metabolite Gene Clusters Analysis); 76 genes, which identified genes associated with secondary metabolism. The results highlighted a well-annotated genome enriched with miscellaneous functional elements related to metabolic processes, pathogenicity, and secondary metabolite biosynthesis (Figure 4; Table 4).

**TABLE 3:** BUSCO (Benchmarking Universal Single-Copy Orthologs) analysis for completeness of genome.

Completeness of genome		
Features	Number	Percentage
Complete BUSCOs (C)	752	99.21
Complete and single-copy BUSCOs (S)	58	7.65
Complete and duplicated BUSCOs (D)	694	91.56
Fragmented BUSCOs (F)	1	0.13
Missing BUSCOs (M)	5	0.66
Total BUSCO groups searched	758	-

**TABLE 4:** Gene prediction and functional annotation.

Functional annotation	Count
Universal Protein Resource (UniProt)	30,579
Cluster of orthologous gene (COG)	21,001
Carbohydrate degrading enzyme (CAZyme)	16,779
Pathogen host interaction database (PHI)	2,937
Gene ontology (GO)	44,376
Kyoto Encyclopedia of Genes and Genomes (KEGG)	433
Antismash	76
Protein families (Pfam)	209,329

### 3.2 Functional analysis

**FIGURE 5:**
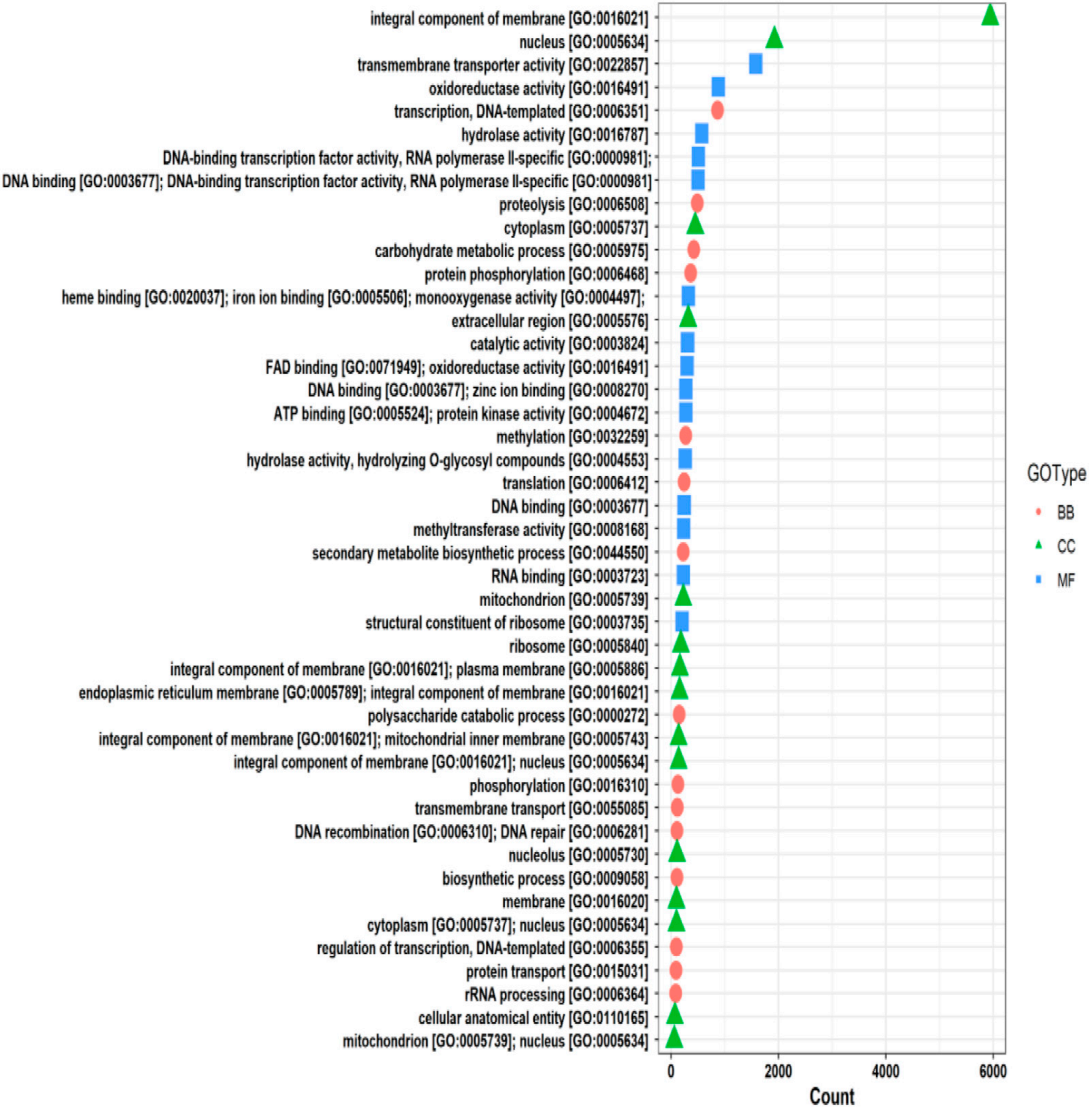
Gene Ontology (GO) classifications of unigenes.

The functional annotation of the *F. oxysporum* f. sp. *lentis* genome revealed that the highest number of hits were detected against the Pfam database, followed by Gene Ontology (GO) and UniProt databases (Figure 4). To further examine the functional classification of genes, 35,458 unigenes were exposed to GO analysis. The unigenes were organized into 44,376 functional groups across three main GO categories, i.e., cellular component, molecular function, and biological process (Figure 5).

**FIGURE 6:**
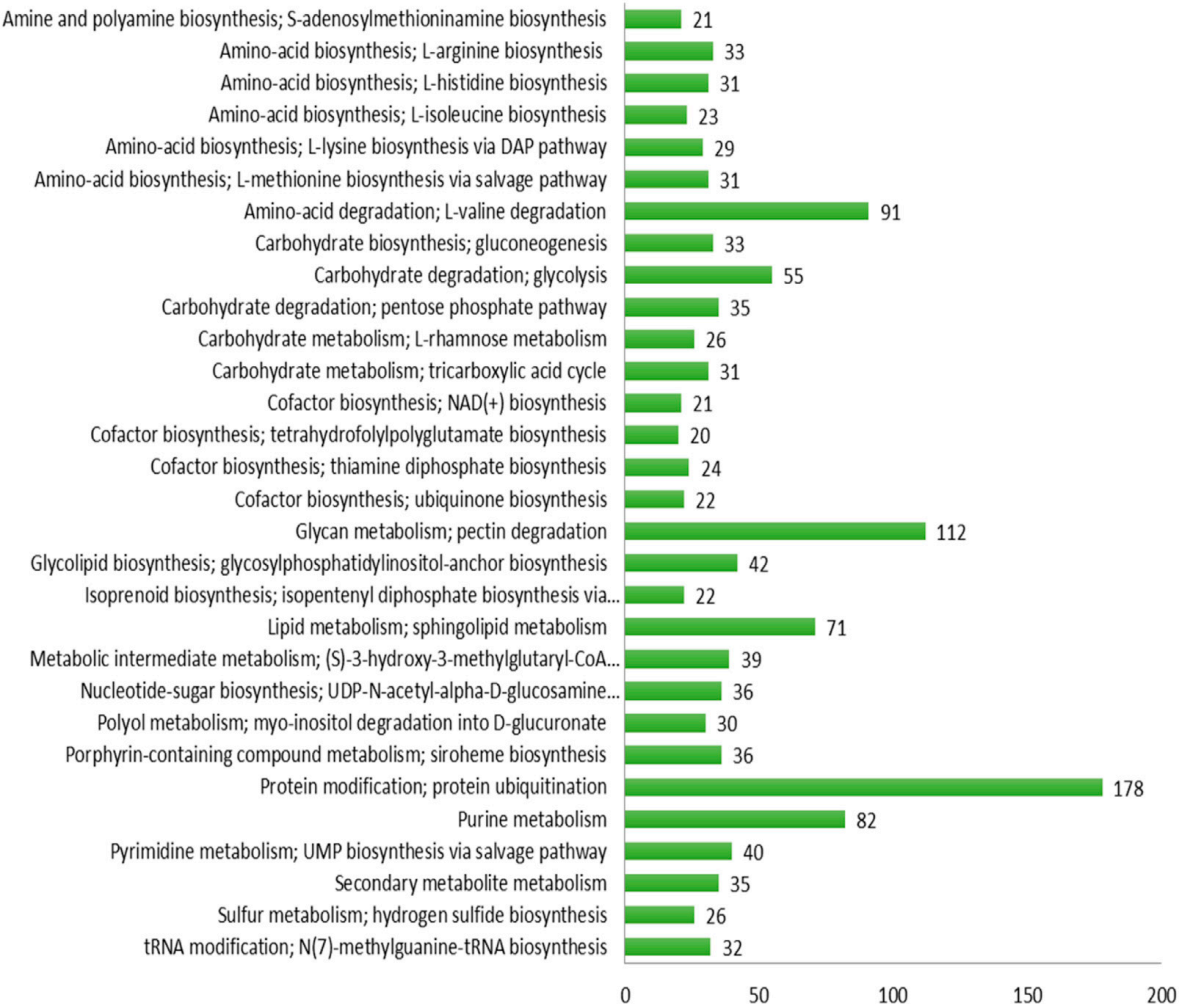
KEGG: Pathway analysis.

**FIGURE 7:**
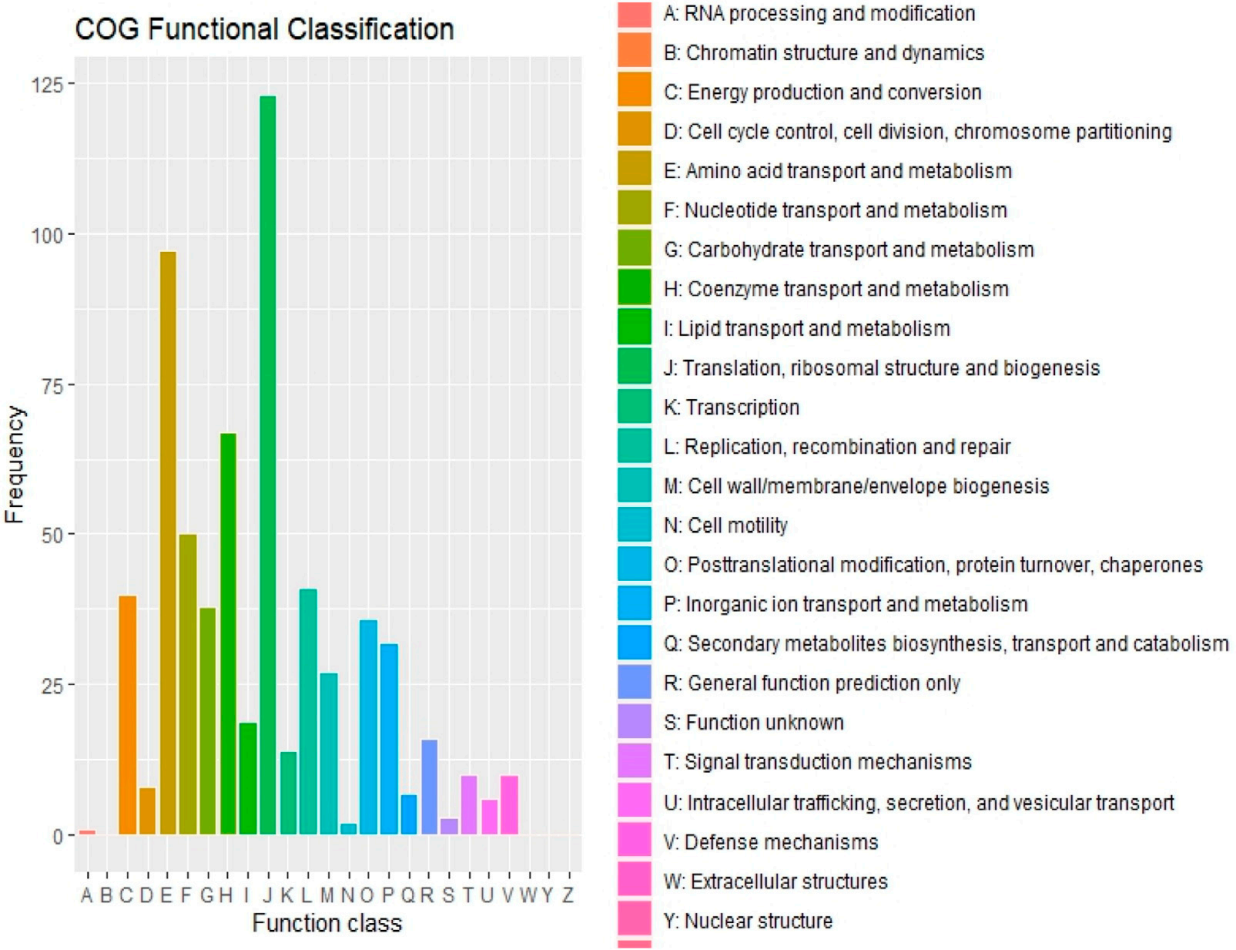
Cluster of Orthologous Genes and their Functional Classification.

**FIGURE 8:**
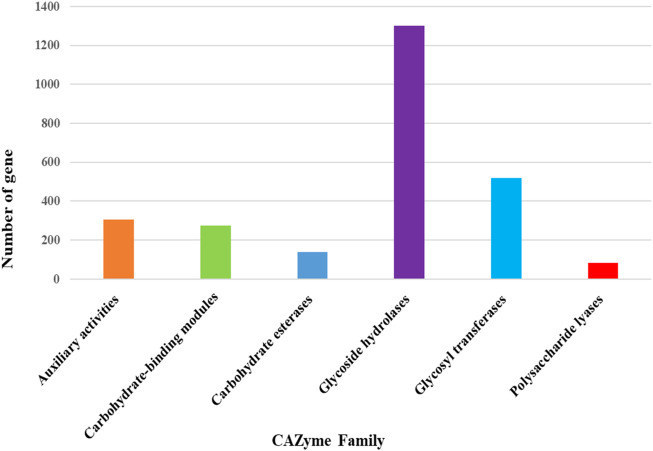
Distribution of CAZyme family putative genes in different classes.

The maximum number of hits was examined against the Pfam database, followed by the GO and UniProt database (Figure 4). To analyze the roles of all unigenes above 35,458 unigenes were used for GO analysis. These unigenes were classified into 44,376 functional groups in three main classes (cellular component, molecular function, and biological process) (Figure 5). The GO annotation results revealed that terms of integral component of membrane, nucleus cytoplasm, and extracellular region were dominant in the cellular component, those of transmembrane transporter activity oxidoreductase activity, binding and catalytic activity were key in molecular function, and those of transcription, proteolysis, carbohydrate metabolic process, protein phosphorylation, and methylation were foremost in biological process (Figure 5). To gain deeper insights into physiological processes, the KOBAS software was utilized to analyze the statistical enrichment of DEGs (Differentially Expressed Genes) within KEGG pathways. This analysis revealed that 433 unigenes were mapped to 107 KEGG pathways. Most of the correlative genes were differently expressed for protein modification and ubiquitination, glycan metabolism (pectin degradation), amino acid degradation (L-valine degradation), lipid metabolism (sphingolipids metabolism), ubiquinone, other amino acid biosynthesis, carbohydrate biosynthesis and metabolism and glycolipid biosynthesis and nucleotide-sugar biosynthesis and sulfur metabolism. Among 107 pathways, the largest pathway was protein modification (178), amino acid degradation (91) and glycan metabolism (112 genes), followed by glycolysis/gluconeogenesis (33 genes), lipid metabolism (71 genes) and purine metabolism (82 genes) (Figure 6). The results of this study showed that all unigenes were involved in development and metabolism indicating that these unigenes much more likely play important roles for fungal developmental stage. The COG functional classification showed that 21,001 unigene were distributed across 25 COG categories (Figure 7). It also showed that general function prediction only accounts for the largest percentage followed by translation, ribosomal structure, and biogenesis, amino acid transport and metabolism, and coenzyme transport and metabolism. Many unigenes were also involved in cluster transcription and signal transduction mechanisms. These results indicated that genes in the above categories play important roles in development. The Fusarium genome sequence was analyzed to determine the presence of Carbohydrate-Active enzymes (CAZymes). The analysis revealed 16,779 putative genes encoding CAZymes, which were distributed across 91 CAZyme protein families. CAZyme are proteins with polysaccharide-degrading enzymatic activities on polysaccharides. We identified 16,779 putative genes that encode CAZyme in the genome of Fusarium. Of the six CAZyme classes, Carbohydrate Esterases (CE), Glycoside Hydrolases (GH), and Polysaccharide Lyases (PL) are Plant Cell Wall Degrading Enzymes (PCWDEs). There were with 306, 274, 518, 82, 1,300, and 139 genes identified in the Auxiliary Activities (AA), Carbohydrate-Binding Molecules (CBM), Glycoside Hydrolases (GT), Polysaccharide Lyases (PL), Glycoside Hydrolases (GH) and Carbohydrate Esterases (CE) classes, respectively (Figure 8) (Table 5).

**TABLE 5:** Distribution of different classes of Carbohydrate-Active Enzymes (CAZyme**)**.

Carbohydrate-active enzymes (CAZyme)	Total number
Plant cell wall degrading enzymes (PCWDEs)	
Polysaccharide Lyases (PL)	82
Glycoside Hydrolases (GH)	1,301
Carbohydrate Esterases (CE)	139
Other CAZyme	
Auxiliary Activities (AA)	306
Carbohydrate-Binding Molecule (CBM)	274
glycosyltransferases (GT)	518

**FIGURE 9:**
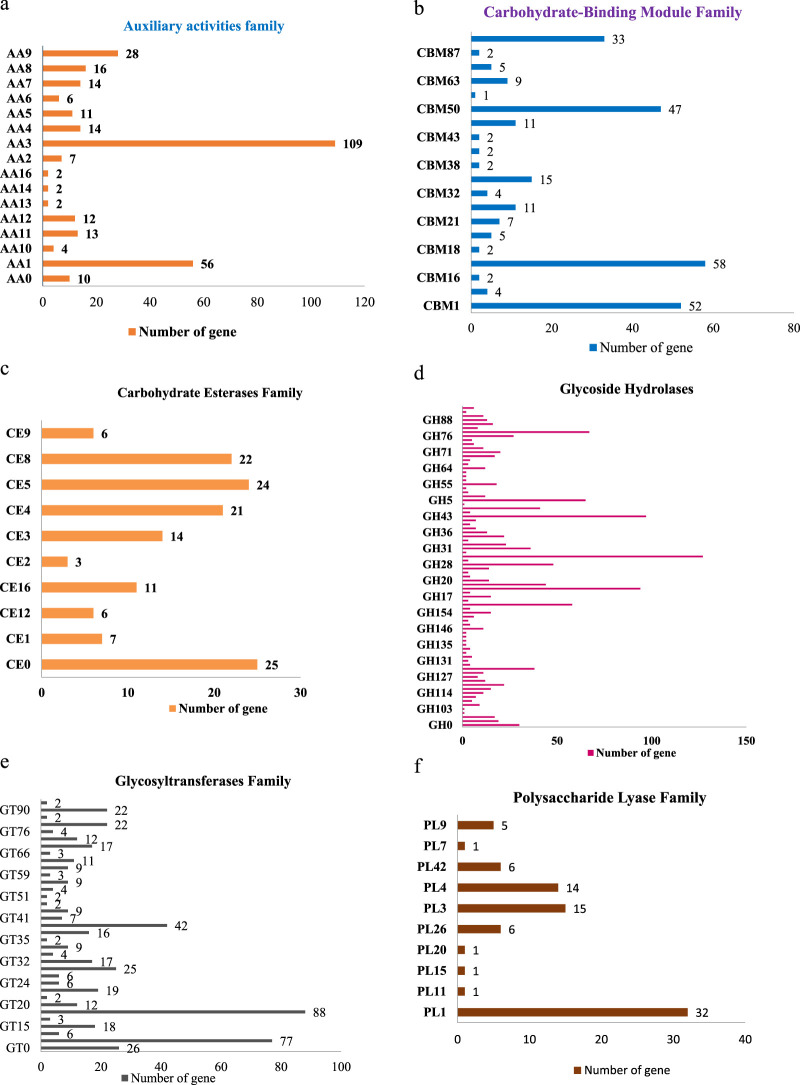
**(a)** Distribution of CAZyme family putative genes in Auxiliary activities (AA). **(b)** Distribution of CAZyme family putative genes in Carbohydrate-Binding Module (CBM) Family. **(c)** Distribution of CAZyme family putative genes in Carbohydrate Esterases (CE) Family. **(d)** Distribution of CAZyme family putative genes in Glycoside Hydrolases (GH) Family. **(e)** Distribution of CAZyme family putative genes in Glycosyltransferases (GT) Family. **(f)** Distribution of CAZyme family putative genes in Polysaccharide Lyase (PL) Family.

This includes 306 Auxiliary Activities (AAs) [Figure 9a; Supplementary Tables S1a–f], 274 Carbohydrate-Binding Modules (CBMs) [Figure 9b; Supplementary Tables S1a–f], 139 Carbohydrate Esterases (CEs) [Figure 9c; Supplementary Tables S1a–f], 1,301 Glycoside Hydrolases (GHs) [Figure 9d; Supplementary Tables S1a–f], 518 Glycosyltransferases (GTs) [Figure 9e; Supplementary Tables S1a–f] and 82 Polysaccharide Lyases (PLs) [Figure 9f; Supplementary Tables S1a–f]. The predictions suggested more GHs than GTs in the Fusarium genome. GHs (glycosidases or glycosyl hydrolases, EC 3.2.1.) are enzymes that catalyze the hydrolysis of glycosidic bonds of complex carbohydrates and key enzymes involved in carbohydrate metabolism. In addition, GHs are common enzymes in nature that degrade the most abundant biomasses, such as cellulose, hemicellulose, and starch.

The genome assembly comprised 12,366 scaffolds, totaling approximately 114 million base pairs (Mb). Using Tantan within the Funannotate pipeline, 4.53% of the genome was identified as repetitive elements and soft-masked accordingly. These repetitive sequences, which include various transposable elements, were preserved in lowercase format to maintain sequence integrity for downstream analyses. The soft-masking approach facilitated accurate gene annotation by preventing misannotation within repetitive regions. Additionally, the identification of these elements provides insights into genome evolution and stability, offering a foundation for comparative genomics and further functional analysis aimed at improving genome annotation accuracy.

Genome annotation using *tRNAscan-SE* within the Funannotate pipeline identified 598 valid tRNA genes. All predicted tRNAs were non-overlapping, supporting the accuracy of gene model predictions and minimizing the risk of annotation artifacts. These tRNA genes, essential for the translational machinery, contribute to the fidelity of protein synthesis by delivering specific amino acids to ribosomes. Their distinct organization likely reflects a stable genome architecture and functional conservation. The relatively high number of tRNA genes suggests a well-structured genome with potential implications for codon usage bias, gene regulation, and evolutionary adaptation.

### 3.3 Pathogen host interactions

**FIGURE 10:**
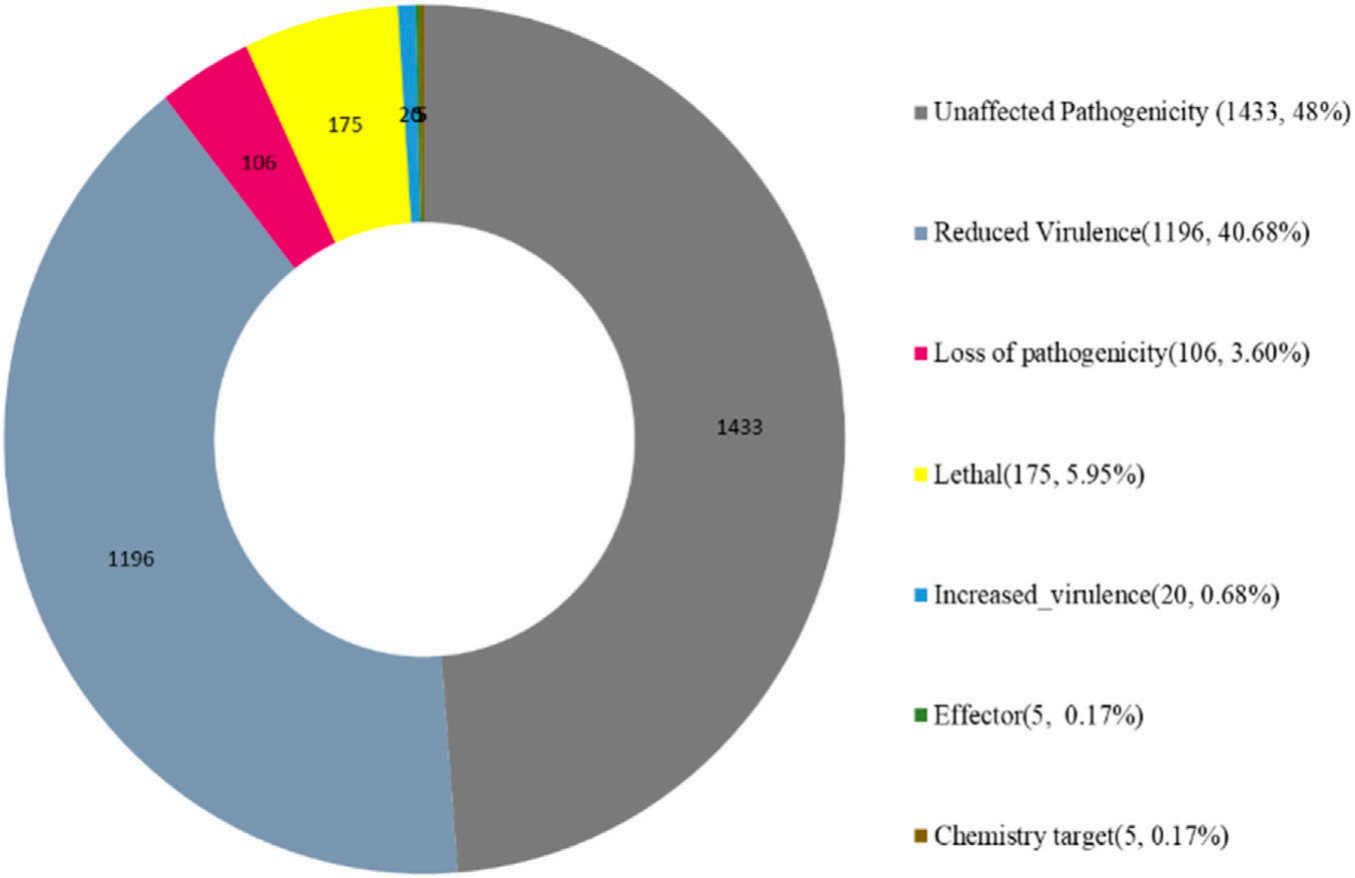
Functions of PHI proteins.

The Pathogen Host Interactions database (PHI-base) has manually curated experimentally verified pathogenicity, virulence, and effector genes from fungal, bacterial, and protist pathogens. The amino acid sequence of the target species of *Fusarium* isolate was compared with the PHI database by using the BLAST software, and the gene of the target species was combined with the functional annotation information to obtain an annotation result (Figure 10). *Fusarium* strain harbors abundant PHI-base genes, including reduced virulence (1,196), increased virulence (hypervirulence) (20), loss of pathogenicity (106), lethal (175), unaffected pathogenicity (1,433), chemical target (5), effector (plant avirulence determinant) (5) and. Reduced virulence and unaffected pathogenicity are the major annotation genes, suggesting that *Fusarium* strain is not a highly pathogenic strain (Figure 10).

### 3.4 Analysis of secondary metabolite biosynthetic gene clusters

**FIGURE 11:**
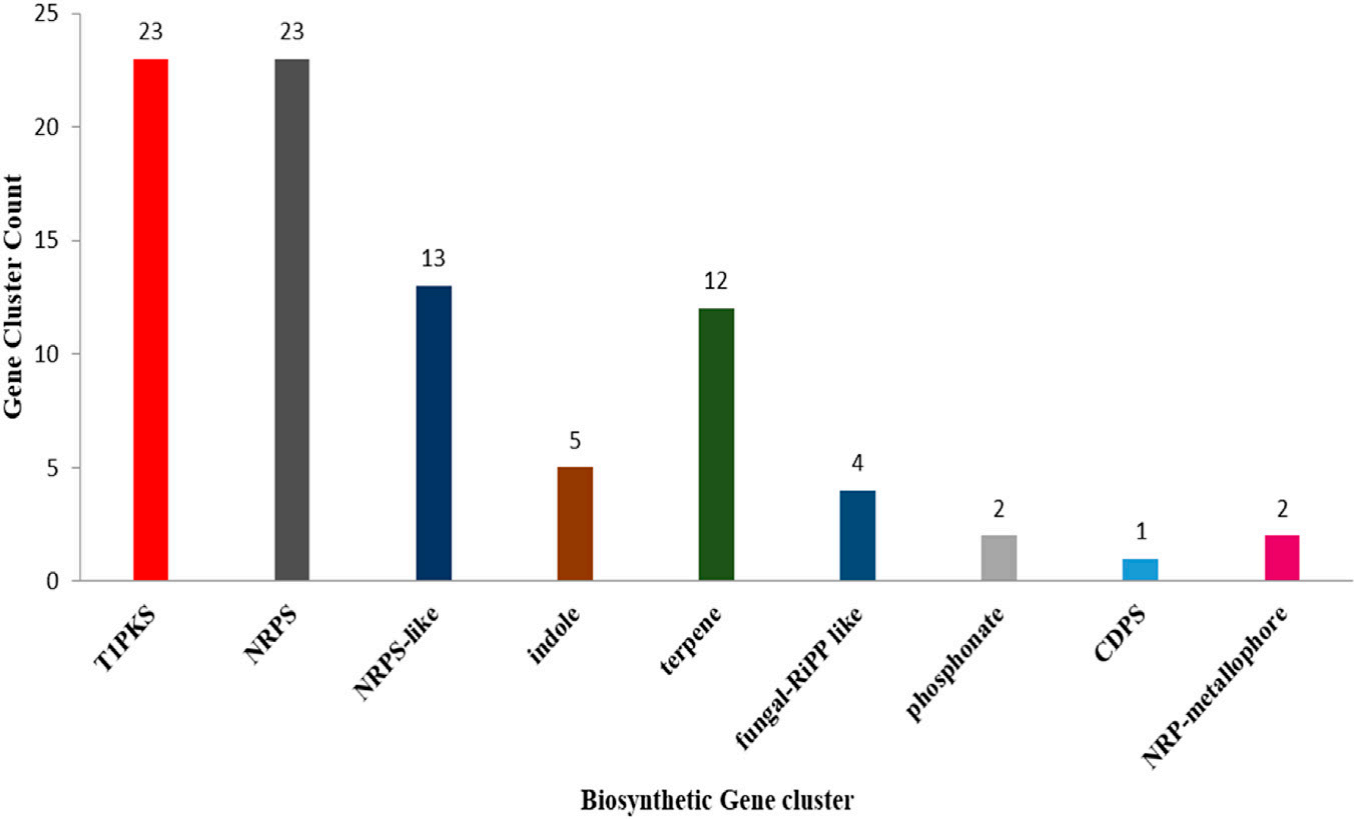
Graphical representation of *Fusarium* fungal genomes revealing putative natural products of many different types.

**FIGURE 12:**
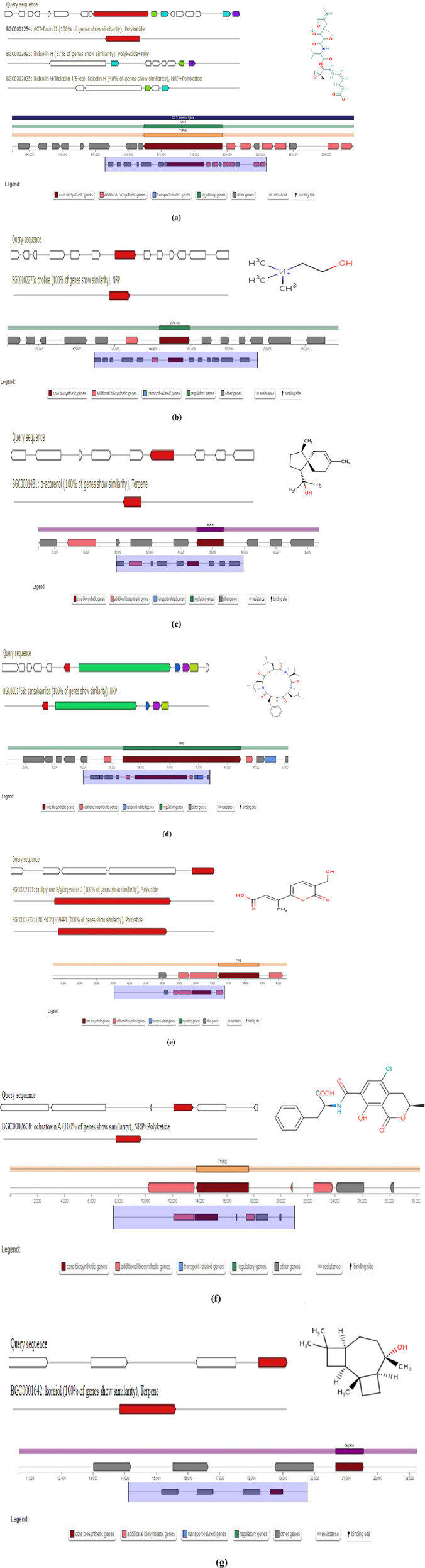
**(a)** AntiSMASH Report; ACT-toxin II: Region 24.1. **(b)** AntiSMASH Report; choline: Region 26.1. **(c)** AntiSMASH Report; α-acorenol; Region 280.1. **(d)** AntiSMASH Report; sansalvamide: Region 362.1. **(e)** AntiSMASH Report; Prolipyrone B: Region 363.1. **(f)** AntiSMASH Report; Ochratoxin A: Region 655.1. **(g)** AntiSMASH Report; Koraiol: Region 873.1.

AntiSMASH analysis suggested that *Fusarium* strain possesses 77 Secondary Metabolite (SM) biosynthetic gene clusters (BGCs), including 23 T1PKS, 23 NRPS, 13 NRPS-like, 5 indole, 12 terpene, 4 fungal-RiPP like, 2 phosphonate, 1 CDPS, 2 NRP-metallophore biosynthetic genes (Figure 11) (Table 6). Only 30% of these BGCs showed gene homologies with known clusters in the MIBiG database. By further comparison with the gene sequences of other reference strains, several BGCs of the *Fusarium* strain with high similarity were identified and predicted to be responsible for the biosynthesis of ACT-Toxin II (Polyketide) in region 24.1, choline (NRPS-like) in region 26.1, α-acorenol (terpene) in region 280.1, Sansalvamide (NRPS) in region 362.1, Prolipyrone B (T1PKS) in region 363.1, Ochratoxin A (NRP + Polyketide) in region 655.1, Karaiol (Terpene) in region 873.1(Figures 12a–g).

**TABLE 6:** Identification of Secondary metabolite (SM) biosynthetic gene clusters (BGCs) using AntiSMASH.

Region	Type	From	To	Most similar known cluster		Similarity
Region 1.1	NRPS-like,NRPS	580,099	662,591			
Region 1.2	NRPS-like	10,08,189	10.51,370			
Region 5.1	T1PKS,NRPS, indole	175,956	235,880			
Region 5.2	NRPS-like	415,181	458,551			
Region 17.1	indole	334,995	356,202			
Region 17.2	terpene	446,737	470,479			
Region 18.1	T1PKS,NRPS	238,666	290,525	lucilactaene	Polyketide	38%
Region 21.1	NRPS-like	304,113	347,104			
Region 23.1	terpene	39,577	63,043			
Region 24.1	NRPS,T1PKS	187,390	239,254	ACT-Toxin II	Polyketide	100%
Region 24.2	fungal-RiPP-like	240,012	300,543			
Region 26.1	NRPS-like	125,882	169,819	choline	NRP	100%
Region 27.1	terpene	50,968	72,134			
Region 28.1	NRPS	305,755	355,162	beauvericin	NRP	20%
Region 28.2	indole	385,359	406,649			
Region 31.1	NRPS	272,317	319,524			
Region 32.1	phosphonate	23,456	45,495	fosfonochlorin	Other	53%
Region 34.1	T1PKS	186,811	233,859	oxyjavanicin	Polyketide	87%
Region 38.1	terpene	105,311	127,302			
Region 41.1	terpene	54,125	75,390			
Region 42.1	NRPS	22,153	69,950			
Region 42.2	T1PKS	305,436	336,780			
Region 45.1	NRPS	279,080	322,830			
Region 47.1	NRPS,T1PKS	154,350	207,897			
Region 51.1	NRPS-like	177,870	221,199			
Region 52.1	T1PKS,NRPS	181,110	233,365	equisetin	NRP + Polyketide	45%
Region 57.1	T1PKS,NRPS	1	72,419	gibepyrone-A	Polyketide	40%
Region 58.1	T1PKS	91,497	138,970			
Region 59.1	T1PKS	39,837	87,897			
Region 59.2	terpene	231,062	254,115	gibberellin	Terpene	71%
Region 62.1	T1PKS,NRPS-like	10,382	91,184	fusaric acid	Polyketide	70%
Region 63.1	terpene	237,401	258,581	squalestatin S1	Terpene	40%
Region 65.1	NRPS-like	84,011	127,272	fusaridione A/(3R,5S)-5-(4-hydroxybenzyl)-3-methyl-3-((2E,4E,6E,8E,10E)-4,8,10-trimethyldodeca-2,4,6,8,10-pentaenoyl)pyrrolidine-2,4-dione	NRP + Polyketide	12%
Region 65.2	T1PKS	143,852	192,421	bikaverin	Polyketide	71%
Region 68.1	T3PKS	182,438	223,946			
Region 70.1	NRPS	136,142	192,369			
Region 79.1	NRPS, indole	22,969	68,606	hexadehydroastechrome/terezine-D/astechrome	NRP	62%
Region 85.1	betalactone	118,437	151,049			
Region 96.1	fungal-RiPP-like	1	59,460			
Region 106.1	fungal-RiPP-like	64,135	124,952			
Region 119.1	fungal-RiPP-like	38,069	99,073			
Region 120.1	NRPS-like	1	28,117			
Region 128.1	T1PKS	15,209	62,807	fujikurin A/fujikurin B/fujikurin C/fujikurin D	Polyketide	50%
Region 132.1	T3PKS	61,112	102,584			
Region 134.1	T1PKS	26,044	96,156	lijiquinone	Polyketide	25%
Region 142.1	T1PKS	73,960	111,514			
Region 148.1	NRPS-like	68,990	107,153			
Region 158.1	phosphonate	18,805	29,514			
Region 175.1	NRPS-like	15,569	60,582			
Region 181.1	NRPS,NRPS-like	1	62,610	fusatrixin/fusapentaxin/fusaoctaxin A	NRP	87%
Region 182.1	NRPS	10,442	66,786			
Region 195.1	CDPS	29,994	50,940			
Region 216.1	NRPS	1	30,852	metachelin C/metachelin A/metachelin A-CE/metachelin B/dimerumic acid 11-mannoside/dimerumic acid	NRP	62%
Region 236.1	NRP-metallophore,NRPS	19,844	72,080			
Region 280.1	terpene	44,998	62,882	α-acorenol	Terpene	100%
Region 281.1	NRP-metallophore,NRPS	4,596	62,843			
Region 287.1	T1PKS	1,825	48,905			
Region 292.1	T1PKS	6,793	55,084	gibepyrone-A	Polyketide	40%
Region 302.1	NRPS	1	34,391			
Region 323.1	T1PKS	1	34,683			
Region 327.1	terpene	1	13,051			
Region 344.1	terpene	1	12,198			
Region 362.1	NRPS	1,901	51,039	sansalvamide	NRP	100%
Region 363.1	T1PKS	22,740	51,020	prolipyrone B/gibepyrone D	Polyketide	100%
Region 371.1	terpene	29,965	49,947	squalestatin S1	Terpene	40%
Region 385.1	NRPS-like	1	25,723			
Region 421.1	NRPS	1	44,364			
Region 468.1	NRPS-like	1	33,044			
Region 567.1	T1PKS	1	34,398			
Region 638.1	NRPS	1	31,118			
Region 655.1	T1PKS	1	30,583	ochratoxin A	NRP + Polyketide	100%
Region 747.1	T1PKS	1	27,057	cyclosporin	NRP	20%
Region 788.1	NRPS,T1PKS	1	25,732	lucilactaene	Polyketide	53%
Region 866.1	indole	1,137	22,294			
Region 873.1	terpene	10,670	23,250	koraiol	Terpene	100%
Region 933.1	NRPS	1	21,814			

AntiSMASH analysis showed that genes within the region 26.1 had a significant BLAST hit with the choline BGC (GenBank: NW_022194793) from *Fusarium proliferatum* ET1 genome assembly (3400500–3441734). BGC region 280.1 of *Fusarium* strain displayed significant similarity with that of α-acorenol (GenBank: CM000599 & NC_030996) from *F. oxysporum* f. sp*. lycopersici* (127,387–148652). Sansalvamide, a cyclic pentadepsipeptide with a potent anticancer effect, was originally isolated from one marine *Fusarium* species. The chemical structure of sansalvamide, with four proteogenic amino acids and one hydroxyl acid, suggested that it could be synthesized by a five-module NRPS where each of the modules would be responsible for incorporating one of the amino acids. BGC region 873.1 of *Fusarium* strain displayed significant similarity with that of Koraiol (GenBank: CM000600 & NC_030997) from *F. oxysporum* f. sp. *lycopersici* (1,264,684–1286383). BGC region 24.1 of the *Fusarium* strain displayed significant similarity with that of ACT-Toxin II (GenBank: NW_022194804) from *F. proliferatum* ET1 genome (252,933–304792).

**FIGURE 13:**
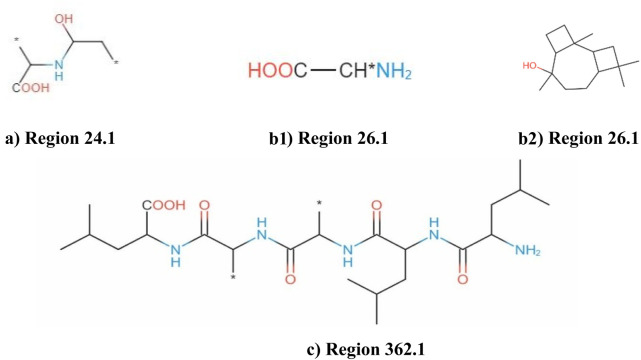
AntiSMASH analysis showing identification of putative compounds biosynthesized by different gene clusters **(a, b1, b2, c)** NRPs and PKSs, NRPS-like, NRPs, Terpene only.

Based on the antiSMASH analysis, compounds **a** were putatively biosynthesized by various NRPS & PKSs, compound b was putatively synthesized from NRPS-like while compounds **c** was plausible products of the NRPS only, compound d was a plausible product of the Terpene only (Figure 13). BGC region 24.1 possesses several additional enzymes including ilicicolin H biosynthetic gene cluster from *Talaromyces variabilis (*GenBank: MK539848.1) and ilicicolin J and 8-epi-ilicicolin H *Neonectria* sp. *DH2 (*GenBank: RQWH01000002.1). BGC region 24.1 possesses several additional enzymes including UNII-YC2Q1O94PT and gibe-pyrone D biosynthetic gene cluster from *Alternaria alternata (*GenBank: AB725683.1).

### 3.5 Synteny analysis

**FIGURE 14:**
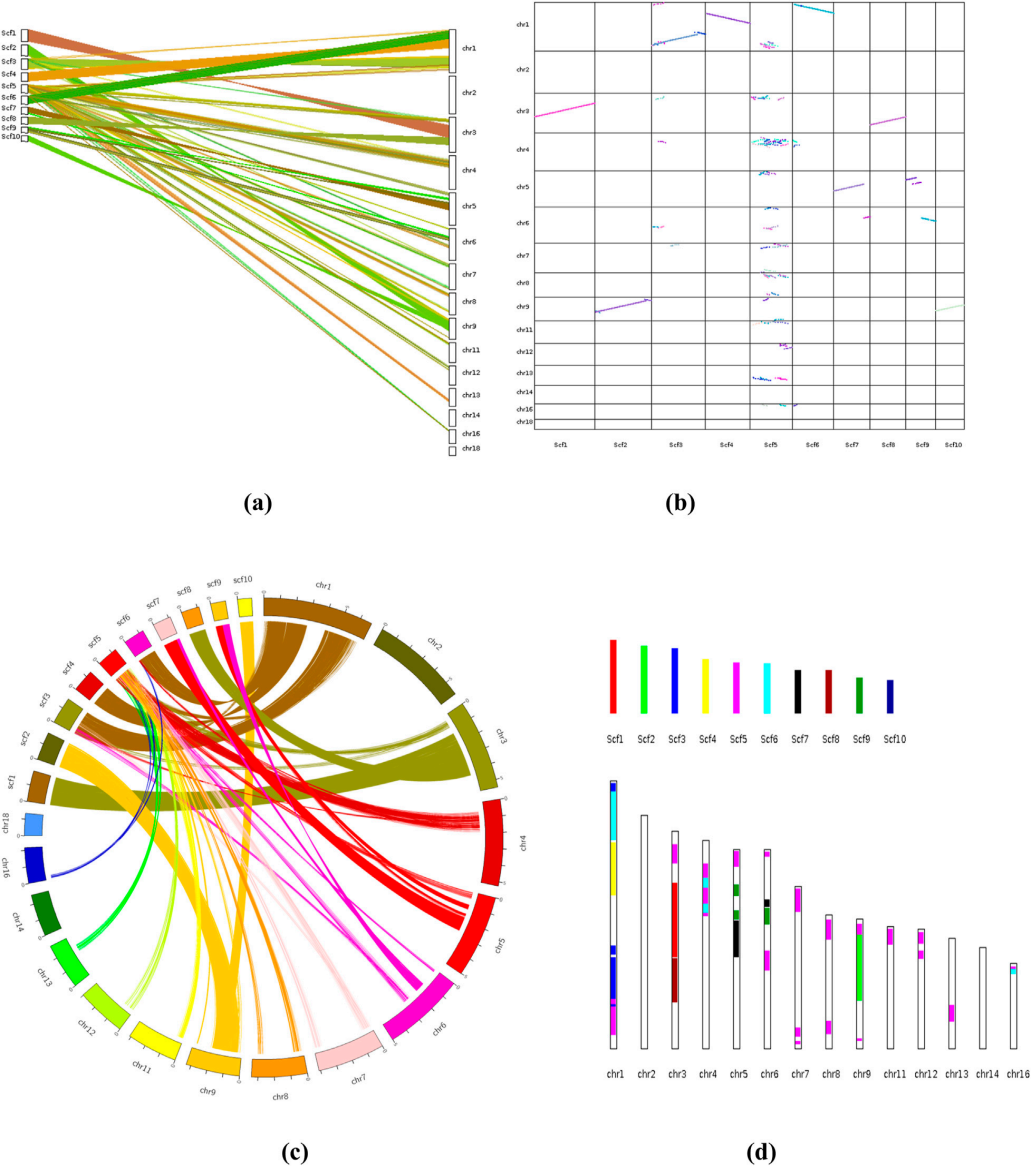
**(a)** Synteny Analysis: Different types of plots showing patterns of synteny and collinearity; [**(a, b)** Dual synteny plot& Dot plot]. **(b)** Chromosomes are labeled “species abbreviation” + ”chromosome ID” in the format. **(c)** Circle plot, **(d)** Bar plot. Sc, *Fusarium oxysporum* strain; chr, *Fusarium oxysporum* f. sp. coaglutinans (Reference species: GCA_014154955.1_SMRT_HiC_Fo5176_genomic).

The synteny analysis was performed to compare the genome sequence of *F. oxysporum* f. sp. *conglutinans* causing Fusarium wilt of cabbage with the draft genome sequence of *Fusarium* assembled in this study. This comparative genomic approach revealed notable collinearity between specific scaffolds in the draft genome and chromosomes in the reference genome. Specifically, scaffold 2 (*scf2*) and scaffold 10 (*scf10*) exhibited strong collinear relationships with chromosome 9 (*chr9*) of *F. oxysporum* f. sp. *conglutinans*, indicating a high level of conserved gene order and orientation across these regions. In addition, scaffold 1 (scf1) and scaffold 8 (scf8) showed substantial collinear blocks with chromosome 3 (chr3) of the reference genome, suggesting evolutionary conservation and potential functional similarities in these genomic regions. Moreover, larger segments of scaffold 3 (scf3), scaffold 4 (scf4), and scaffold 6 (scf6) displayed extensive syntenic blocks with chromosome 2 (chr2) of the reference genome, further supporting the presence of conserved genome architecture between the studied isolate and *F. oxysporum* f. sp. *conglutinans*. These patterns of synteny not only validate the structural integrity of the draft genome but also provide insights into the evolutionary conservation and potential gene function relationships across different *Fusarium* isolates (Figures 14a-d).

## 4 Discussion

In this study, we present the first whole-genome sequencing of *F. oxysporum* f. sp. *lentis*, pathogen responsible for vascular wilt in lentils, using the Illumina Shotgun Sequencing platform. The development of genomic resources is crucial for understanding the genetic basis of pathogenicity and other biological processes in *F. oxysporum* f. sp. *lentis*. The *F. oxysporum* species complex exhibits extensive genetic and functional diversity. Pathogenic strains demonstrate host specificity, whereas non-pathogenic strains do not induce disease symptoms, despite the absence of morphological differences (Nelson et al., 1981; Steinberg et al., 2016). Molecular genetic studies are essential for deciphering pathogenicity mechanisms, which, in turn, are crucial for devicing effective disease management strategies. Our sequencing efforts resulted in a high-quality genome assembly comprising 12,366 contigs with a total assembly length of 124,483,966 bases. BUSCO analysis confirmed 99% completeness, indicating a robust and reliable assembly. The N50 and L50 values were 51,992 and 353, respectively, further indicating a high-quality well-assembled genome.

Functional annotation of the genome revealed 116,998 protein-coding genes, with substantial homology identified across multiple databases, including Uniprot, GO, KEGG, Pfam, COG, CAZyme, PHI, and AntiSMASH. Gene ontology (GO) analysis classified 35,458 unigenes into three major categories: cellular components, molecular functions, and biological processes. Key enriched GO terms included transmembrane transporter activity, oxidoreductase activity, binding, and catalytic activity, suggesting an active role in metabolic and cellular processes. Additionally, 433 unigenes were mapped to 107 KEGG pathways, with significant representation in protein modification and ubiquitination, glycan metabolism, amino acid degradation, lipid metabolism, and carbohydrate biosynthesis.

Carbohydrate-active enzymes (CAZymes) play a pivotal role in fungal pathogenicity by facilitating plant cell wall degradation. We identified 16,779 CAZyme-encoding genes distributed across 91 protein families, with glycoside hydrolases (GH) and glycosyltransferases (GT) being the most abundant classes. The presence of hemicellulose-degrading enzymes (GH10, GH11, GH115) and pectin-degrading enzymes (GH43, GH51, GH78, GH93, PL1, PL3, PL4) underscores the ability of *F. oxysporum* f. sp. *lentis* to efficiently degrade plant cell walls. Interestingly, an expansion of fungal cell wall biosynthesis-related CAZymes was also observed, emphasizing the importance of chitin and β-glucan remodeling in fungal development and host colonization.

Pathogenicity-related gene analysis using the PHI-base database identified multiple genes associated with different pathogenicity levels: reduced virulence (1,196), hypervirulence (20), loss of pathogenicity (106), lethal effects (175), and unaffected pathogenicity (1,433). The predominance of reduced virulence and unaffected pathogenicity genes suggests that while this strain possesses virulence factors, it may not be highly aggressive compared to other pathogenic *F. oxysporum* strains.

The identification of secondary metabolite biosynthetic gene clusters (BGCs) is critical for understanding fungal virulence and toxin production. AntiSMASH analysis revealed 77 secondary metabolite clusters, including 23 Type I polyketide synthase (T1PKS), 23 non-ribosomal peptide synthetases (NRPS), 13 NRPS-like, 5 indole, 12 terpene, 4 fungal RiPP-like, 2 phosphonate, 1 CDPS, and 2 NRP-metallophore biosynthetic genes. These findings lay the groundwork for further investigations into the role of secondary metabolites in pathogenicity and host interactions.

Advancements in sequencing technologies have significantly improved genome assembly quality. While early genome sequencing of *F. oxysporum* relied on Sanger sequencing (Broad Institute, 2007; Ma et al., 2010). However, next-generation sequencing (NGS) platforms such as Illumina and BGI have accelerated genome assembly at reduced cost. Third-generation sequencing technologies, such as Pacific Biosciences (PacBio) and Oxford Nanopore Technologies (ONT), offer longer read lengths, with superior genome assemblies (van Dijk et al., 2018; Amarasinghe et al., 2020; Giani et al., 2020).

## 5 Conclusion

This study presents the first whole-genome sequencing and assembly of *F. oxysporum* f. sp. *lentis* using Illumina short-read sequencing technology. The high-quality genome assembly, with 99% completeness based on BUSCO analysis, provides critical insights into the genetic basis of pathogenicity, carbohydrate-active enzymes (CAZymes), and secondary metabolite biosynthetic gene clusters (BGCs). Functional annotation revealed key gene families associated with virulence, metabolic pathways, and host adaptation, enhancing our understanding of *F. oxysporum* evolution and pathogenic mechanisms. Although this strain harbors significant virulence genes, PHI-base analysis advocates moderate pathogenic potential, underscoring the need for further investigation into host-pathogen interactions. This study offers novel insights into the genetic makeup, virulence factors, and metabolic potential of *F. oxysporum* f. sp. *lentis*, contributing to future research on host-pathogen interactions and disease management strategies and lentil breeding strategies. Future research should prioritize comparative genomics of *F. oxysporum* f. sp. *lentis* isolates to identify race-specific virulence factors and host resistance mechanisms. The integration of long-read sequencing technologies, such as PacBio or Oxford Nanopore, will enhance genome assembly, facilitating the identification of structural variations and transposable elements linked to pathogenicity. Additionally, transcriptomic and proteomic studies will provide functional validation of key effector genes and metabolic pathways involved in lentil infection. Understanding the molecular basis of virulence and secondary metabolism in *F. oxysporum* f. sp. *lentis* will facilitate the development of disease-resistant lentil cultivars and targeted biocontrol strategies to mitigate *Fusarium* wilt.

## Data Availability

The datasets presented in this study can be found in online repositories. The names of the repository/s and accession number(s) can be found at online repositories at NCBI https://www.ncbi.nlm.nih.gov/sra/?term=SRR25383692.
