# Supplementary material for: Whole genome sequencing and functional annotation of Fusarium oxysporum f. sp. lentis to unravel virulence and secondary metabolite biosynthesis gene clusters
Source: Front Genet. 2025 Jun 18;16:1585510. doi: 10.3389/fgene.2025.1585510 (PMC12213878; doi:10.3389/fgene.2025.1585510)
Supplement: Supplementary file 1 [file Table1.docx]

**Table s1 (a):** **Annotation of putative genes identified in carbohydrate-active enzymes (CAZymes) in Auxiliary Activities (AA)**

| **CAZyme family** | **Annotation** | **Copy number** |
| --- | --- | --- |
| **Auxiliary activities (AA)** | | |
| AA0 | - | 10 |
| AA1 | [Laccase / p-diphenol:oxygen oxidoreductase / ferroxidase (EC 1.10.3.2)](http://www.enzyme-database.org/query.php?ec=1.10.3.2) | 56 |
| AA10 | [AA10 (formerly CBM33) proteins are copper-dependent lytic polysaccharide monooxygenases (LPMOs); some proteins have been shown to act on chitin, others on cellulose; lytic cellulose monooxygenase (C1-hydroxylating) (EC 1.14.99.54)](http://www.enzyme-database.org/query.php?ec=1.14.99.54) | 4 |
| AA11 | [copper-dependent lytic polysaccharide monooxygenases (LPMOs); cleavage of chitin chains with oxidation of C-1 has been demonstrated for a AA11 LPMO from Aspergillus oryzae;lytic chitin monooxygenase (1.14.99.53)](http://www.enzyme-database.org/query.php?ec=1.14.99.53) | 13 |
| AA12 | pyrroloquinoline quinone-dependent oxidoreductase activity | 12 |
| AA13 | [copper-dependent lytic polysaccharide monooxygenases (LPMOs); cleavage of starch with oxidation of C-1 at the site of cleavage has been demonstrated for the LPMO encoded by gene NCU08746 from Neurospora crassa; lytic starch monooxygenase / starch oxidase (glycosidic bond-cleaving) (EC 1.14.99.55)](http://www.enzyme-database.org/query.php?ec=1.14.99.55) | 2 |
| AA14 | [copper-dependent lytic polysaccharide monooxygenases (LPMOs); cleavage of xylan with oxidation of C-1 has been demonstrated for two AA14 LPMOs from Trametes coccinea (Pycnoporus coccineus); lytic xylan monooxygenase / xylan oxidase (glycosidic bond-cleaving) (EC 1.14.99.-)](http://www.enzyme-database.org/query.php?ec=1.14.99.*) | 2 |
| AA16 | [Lytic cellulose monooxygenase (C1-hydroxylating) (EC 1.14.99.54)](http://www.enzyme-database.org/query.php?ec=1.14.99.54) | 2 |
| AA2 | manganese peroxidase (EC 1.11.1.13); versatile peroxidase (EC 1.11.1.16); lignin peroxidase (EC 1.11.1.14); peroxidase (EC 1.11.1.-); cytochrome-c peroxidase (EC 1.11.1.5); ascorbate peroxidase (EC 1.11.1.11) | 7 |
| AA3 | cellobiose dehydrogenase (EC 1.1.99.18); glucose 1-oxidase (EC 1.1.3.4); aryl alcohol oxidase (EC 1.1.3.7); | 109 |
| AA4 | [vanillyl-alcohol oxidase (EC 1.1.3.38)](http://www.enzyme-database.org/query.php?ec=1.1.3.38) | 14 |
| AA5 | Oxidase with oxygen as acceptor (EC 1.1.3.-); galactose oxidase (EC 1.1.3.9); glyoxal oxidase (EC 1.2.3.15); alcohol oxidase (EC 1.1.3.13); raffinose oxidase (EC 1.1.3.-) | 11 |
| AA6 | [1,4-benzoquinone reductase (EC. 1.6.5.6)](http://www.enzyme-database.org/query.php?ec=1.6.5.6) | 6 |
| AA7 | glucooligosaccharide oxidase (EC 1.1.3.-); chitooligosaccharide oxidase (EC 1.1.3.-); cellooligosaccharide dehydrogenase (EC 1.1.99.-) | 14 |
| AA8 | Iron reductase domain | 16 |
| AA9 | copper-dependent lytic polysaccharide monooxygenases (LPMOs); cleavage of cellulose chains with oxidation of carbons C1 and/or C4 and C-6); lytic cellulose monooxygenase (C1-hydroxylating) (EC 1.14.99.54); lytic cellulose monooxygenase (C4-dehydrogenating) (EC 1.14.99.56) | 28 |
| **Total** | | **306** |

**Table s1 (b):** **Annotation of putative genes identified in Carbohydrate-Active enzymes (CAZymes) in Carbohydrate Binding Modules (CBM)**

| **CAZyme family** | **Annotation** | **Copy number** |
| --- | --- | --- |
| **Carbohydrate binding modules (CBM)** | | |
| CBM1 | non-hydrolytic polysaccharide-binding protein | 52 |
| CBM13 | xylanase | 4 |
| CBM16 | cellulose and glucomannan | 2 |
| CBM18 | chitin-binding function | 58 |
| CBM18 | chitin-binding function | 2 |
| CBM20 | granular starch-binding function | 5 |
| CBM21 | granular starch-binding function | 7 |
| CBM24 | α-1,3-glucan (mutan)-binding function | 11 |
| CBM32 | galactose and lactose | 4 |
| CBM35 | Cellvibrio xylan-degrading enzymes | 15 |
| CBM38 | inulin-binding function | 2 |
| CBM42 | arabinofuranose (present in arabinoxylan) | 2 |
| CBM43 | β-1,3-glucan binding function | 2 |
| CBM48 | glycogen-binding function | 11 |
| CBM50 | chitopentaose | 47 |
| CBM6 | cellulose-binding function | 1 |
| CBM63 | expansin EXLX1 has been shown to bind cellulose | 9 |
| CBM67 | L-rhamnose binding activity | 5 |
| CBM87 | α-1,4-N-acetylgalactosamine-rich regions of galactosaminogalactan | 2 |
| CBM91 | Binding to xylans | 33 |
| **Total** | | **274** |

**Table 6 (c): Annotation of putative genes identified in Carbohydrate-Active enzymes (CAZymes) in Carbohydrate Esterases (CE)**

| **CAZyme family** | | **Annotation** | **Copy number** | |
| --- | --- | --- | --- | --- |
| **Carbohydrate esterases (CE)** | | | | |
| CE0 |  | | | 25 |
| CE1 | acetyl xylan esterase (EC 3.1.1.72); cinnamoyl esterase (EC 3.1.1.-); feruloyl esterase (EC 3.1.1.73) | | | 7 |
| CE12 | pectin acetylesterase (EC 3.1.1.-); rhamnogalacturonan acetylesterase (EC 3.1.1.-); acetyl xylan esterase (EC 3.1.1.72) | | | 6 |
| CE16 | [acetylesterase (EC 3.1.1.6) active on various carbohydrate acetyl esters](http://www.enzyme-database.org/query.php?ec=3.1.1.6) | | | 11 |
| CE2 | [acetyl xylan esterase (EC 3.1.1.72)](http://www.enzyme-database.org/query.php?ec=3.1.1.72) | | | 3 |
| CE3 | [acetyl xylan esterase (EC 3.1.1.72)](http://www.enzyme-database.org/query.php?ec=3.1.1.72) | | | 14 |
| CE4 | acetyl xylan esterase (EC 3.1.1.72); chitin deacetylase (EC 3.5.1.41); chitooligosaccharide deacetylase (EC 3.5.1.-) | | | 21 |
| CE5 | acetyl xylan esterase (EC 3.1.1.72); cutinase (EC 3.1.1.74) | | | 24 |
| CE8 | [pectin methylesterase (EC 3.1.1.11)](http://www.enzyme-database.org/query.php?ec=3.1.1.11) | | | 22 |
| CE9 | N-acetylglucosamine 6-phosphate deacetylase (EC 3.5.1.25); N-acetylgalactosamine 6-phosphate deacetylase (EC 3.5.1.80) | | | 6 |
| **Total** | | | | 139 |

**Table 6 (d): Annotation of putative genes identified in Carbohydrate-Active enzymes (CAZymes) in Glycoside Hydrolases (GH)**

| **CAZyme family** | **Annotation** | **Copy number** |
| --- | --- | --- |
| **Glycoside hydrolases (GH)** | | |
| GH0 |  | 30 |
| GH1 | β-glucosidase (EC 3.2.1.21); β-galactosidase (EC 3.2.1.23); β-mannosidase (EC 3.2.1.25); β-glucuronidase (EC 3.2.1.31); β-xylosidase (EC 3.2.1.37); β-D-fucosidase (EC 3.2.1.38); phlorizin hydrolase (EC 3.2.1.62); exo-β-1,4-glucanase (EC 3.2.1.74); 6-phospho-β-galactosidase (EC 3.2.1.85); | 19 |
| GH10 | endo-1,4-β-xylanase (EC 3.2.1.8); endo-1,3-β-xylanase (EC 3.2.1.32); tomatinase (EC 3.2.1.-); xylan endotransglycosylase (EC 2.4.2.-); endo-β-1,4-glucanase (EC 3.2.1.4) | 17 |
| GH102 | [peptidoglycan lytic transglycosylase (EC 3.2.1.-)](http://www.enzyme-database.org/query.php?ec=3.2.1.*) | 1 |
| GH103 | [peptidoglycan lytic transglycosylase (EC 3.2.1.-)](http://www.enzyme-database.org/query.php?ec=3.2.1.*) | 1 |
| GH105 | unsaturated rhamnogalacturonyl hydrolase (EC 3.2.1.172); d-4,5-unsaturated β-glucuronyl hydrolase (EC 3.2.1.-); d-4,5-unsaturated α-galacturonidase (EC 3.2.1.-) | 9 |
| GH106 | α-L-rhamnosidase (EC 3.2.1.40); rhamnogalacturonan α-L-rhamnohydrolase (EC 3.2.1.174) | 5 |
| GH11 | endo-β-1,4-xylanase (EC 3.2.1.8); exo-1,4-β-xylosidase (EC 3.2.1.-) | 7 |
| GH114 | [endo-α-1,4-polygalactosaminidase (EC 3.2.1.109)](http://www.enzyme-database.org/query.php?ec=3.2.1.109) | 11 |
| GH115 | xylan α-1,2-glucuronidase (3.2.1.131); α-(4-O-methyl)-glucuronidase (3.2.1.-) | 15 |
| GH12 | endoglucanase (EC 3.2.1.4); xyloglucan hydrolase (EC 3.2.1.151); β-1,3-1,4-glucanase (EC 3.2.1.73); xyloglucan endotransglycosylase (EC 2.4.1.207) | 22 |
| GH125 | [exo-α-1,6-mannosidase (EC 3.2.1.163)](http://www.enzyme-database.org/query.php?ec=3.2.1.163) | 12 |
| GH127 | β-L-arabinofuranosidase (EC 3.2.1.185); 3-C-carboxy-5-deoxy-L-xylose (aceric acid) hydrolase (EC 3.2.1.-); α-1,3-(3,6)-anhydro-D-galactosidase (EC 3.2.1.-) | 8 |
| GH128 | β-1,3-glucanase (EC 3.2.1.39); β-1,3-glucosidase (EC 3.2.1.-) | 11 |
| GH13 | α-amylase (EC 3.2.1.1); pullulanase (EC 3.2.1.41); cyclomaltodextrin glucanotransferase (EC 2.4.1.19); cyclomaltodextrinase (EC 3.2.1.54); trehalose-6-phosphate hydrolase (EC 3.2.1.93); oligo-α-glucosidase (EC 3.2.1.10); maltogenic amylase (EC 3.2.1.133); neopullulanase (EC 3.2.1.135); α-glucosidase (EC 3.2.1.20) | 38 |
| GH130 | β-1,4-mannosylglucose phosphorylase (EC 2.4.1.281); β-1,4-mannooligosaccharide phosphorylase (EC 2.4.1.319); β-1,4-mannosyl-N-acetyl-glucosamine phosphorylase (EC 2.4.1.320); β-1,2-mannobiose phosphorylase (EC 2.4.1.339) | 4 |
| GH131 | endo-β-1,4-glucanase (EC 3.2.1.21); exo-β-1,3-glucanase (EC 3.2.1.58) | 3 |
| GH132 | [; β-1,3-glucosidase (EC 3.2.1.-)](http://www.enzyme-database.org/query.php?ec=3.2.1.*) | 5 |
| GH133 | [amylo-α-1,6-glucosidase (EC 3.2.1.33)](http://www.enzyme-database.org/query.php?ec=3.2.1.33) | 2 |
| GH134 | [endo-β-1,4-mannanase (EC 3.2.1.78);](http://www.enzyme-database.org/query.php?ec=3.2.1.78) | 4 |
| GH135 | [α-1,4-galactosaminogalactan hydrolase (EC 3.2.1.-)](http://www.enzyme-database.org/query.php?ec=3.2.1.*) | 2 |
| GH139 | [α-2-O-Me-L-fucosidase (EC 3.2.1.-)](http://www.enzyme-database.org/query.php?ec=3.2.1.*) | 2 |
| GH141 | α-L-fucosidase (EC 3.2.1.51); xylanase (EC 3.2.1.8) | 2 |
| GH142 | [β-L-arabinofuranosidase (EC 3.2.1.185)](http://www.enzyme-database.org/query.php?ec=3.2.1.185) | 2 |
| GH146 | [β-L-arabinofuranosidase (EC 3.2.1.185)](http://www.enzyme-database.org/query.php?ec=3.2.1.185) | 11 |
| GH15 | glucoamylase (EC 3.2.1.3); glucodextranase (EC 3.2.1.70); α,α-trehalase (EC 3.2.1.28); dextran dextrinase (EC 2.4.1.2) | 4 |
| GH15 | glucoamylase (EC 3.2.1.3); glucodextranase (EC 3.2.1.70); α,α-trehalase (EC 3.2.1.28); dextran dextrinase (EC 2.4.1.2) | 3 |
| GH152 | [β-1,3-glucanase (EC 3.2.1.39)](http://www.enzyme-database.org/query.php?ec=3.2.1.39) | 6 |
| GH154 | β-glucuronidase (3.2.1.31); β-1,6-D-glucuronidase (EC 3.2.1.-); β-1,6-D-glucuronidase (EC 3.2.1.-) | 15 |
| GH16 | xyloglucan:xyloglucosyltransferase (EC 2.4.1.207); keratan-sulfate endo-1,4-β-galactosidase (EC 3.2.1.103); endo-1,3-β-glucanase / laminarinase (EC 3.2.1.39); endo-1,3(4)-β-glucanase (EC 3.2.1.6); licheninase (EC 3.2.1.73) | 4 |
| GH16 | xyloglucan:xyloglucosyltransferase (EC 2.4.1.207); keratan-sulfate endo-1,4-β-galactosidase (EC 3.2.1.103); endo-1,3-β-glucanase / laminarinase (EC 3.2.1.39); endo-1,3(4)-β-glucanase (EC 3.2.1.6); licheninase (EC 3.2.1.73) | 58 |
| GH162 | [endo-β-1,2-glucanase (EC 3.2.1.71)](http://www.enzyme-database.org/query.php?ec=3.2.1.71) | 3 |
| GH17 | glucan endo-1,3-β-glucosidase (EC 3.2.1.39); licheninase (EC 3.2.1.73); ABA-specific β-glucosidase (EC 3.2.1.175); β-1,3-glucanosyltransglycosylase (EC 2.4.1.-); β-1,3-glucosidase (EC 3.2.1.-) | 15 |
| GH172 | difructose-anhydride-I synthase (EC 4.2.1.179 (old 3.2.1.134)); α-D-Arabinofuranosidase (EC 3.2.1.-); α-D-Fructofuranosidase (EC 3.2.1.-) | 4 |
| GH18 | chitinase (EC 3.2.1.14); lysozyme (EC 3.2.1.17); endo-β-N-acetylglucosaminidase (EC 3.2.1.96); peptidoglycan hydrolase with endo-β-N-acetylglucosaminidase specificity (EC 3.2.1.-); Nod factor hydrolase (EC 3.2.1.-); xylanase inhibitor; concanavalin B; narbonin; dΙ-N-acetylchitobiase / reducing-end exo-hexosaminidase (EC 3.2.1.-); chitobiosidase (EC 3.2.1.200) | 94 |
| GH2 | β-galactosidase (EC 3.2.1.23) ; β-mannosidase (EC 3.2.1.25); β-glucuronidase (EC 3.2.1.31); α-L-arabinofuranosidase (EC 3.2.1.55); mannosylglycoprotein endo-β-mannosidase (EC 3.2.1.152); exo-β-glucosaminidase (EC 3.2.1.165); α-L-arabinopyranosidase (EC 3.2.1.-); β-galacturonidase (EC 3.2.1.-); β-xylosidase (EC 3.2.1.37); β-D-galactofuranosidase (EC 3.2.1.146); β-glucosidase (EC 3.2.1.21); β-galacturonidase RGII specific (EC 3.2.1.-); glycyrrhizin β-glucuronidase (EC 3.2.1.128) | 44 |
| GH20 | β-hexosaminidase (EC 3.2.1.52); lacto-N-biosidase (EC 3.2.1.140); β-1,6-N-acetylglucosaminidase (EC 3.2.1.-); β-6-SO3-N-acetylglucosaminidase (EC 3.2.1.-) | 14 |
| GH23 | lysozyme type G (EC 3.2.1.17); peptidoglycan lyase (EC 4.2.2.n1) also known in the literature as peptidoglycan lytic transglycosylase; chitinase (EC 3.2.1.14) | 4 |
| GH24 | [lysozyme (EC 3.2.1.17)](http://www.enzyme-database.org/query.php?ec=3.2.1.17) | 3 |
| GH27 | α-galactosidase (EC 3.2.1.22); α-N-acetylgalactosaminidase (EC 3.2.1.49); isomalto-dextranase (EC 3.2.1.94); β-L-arabinopyranosidase (EC 3.2.1.88); galactan:galactan galactosyltransferase (EC 2.4.1.-) | 14 |
| GH28 | polygalacturonase (EC 3.2.1.15); exo-polygalacturonase (EC 3.2.1.67); exo-polygalacturonosidase (EC 3.2.1.82); rhamnogalacturonase (EC 3.2.1.171); rhamnogalacturonan α-1,2-galacturonohydrolase (EC 3.2.1.173); xylogalacturonan hydrolase (EC 3.2.1.-) | 48 |
| GH29 | α-L-fucosidase (EC 3.2.1.51); α-1,3/1,4-L-fucosidase (EC 3.2.1.111); α-1,2-L-fucosidase (EC 3.2.1.63); α-1,6-fucosidase (EC 3.2.1.127); α-L-glucosidase (EC 3.2.1.-) | 3 |
| GH3 | β-glucosidase (EC 3.2.1.21); xylan 1,4-β-xylosidase (EC 3.2.1.37); β-glucosylceramidase (EC 3.2.1.45); β-N-acetylhexosaminidase (EC 3.2.1.52); α-L-arabinofuranosidase (EC 3.2.1.55); glucan 1,4-β-glucosidase (EC 3.2.1.74) | 127 |
| GH30 | endo-β-1,4-xylanase (EC 3.2.1.8); β-glucosidase (3.2.1.21); β-glucuronidase (EC 3.2.1.31); β-xylosidase (EC 3.2.1.37); β-fucosidase (EC 3.2.1.38); glucosylceramidase (EC 3.2.1.45) | 2 |
| GH31 | α-glucosidase (EC 3.2.1.20); α-galactosidase (EC 3.2.1.22); α-mannosidase (EC 3.2.1.24); α-1,3-glucosidase (EC 3.2.1.84); sucrase-isomaltase (EC 3.2.1.48) (EC 3.2.1.10); α-xylosidase (EC 3.2.1.177); α-glucan lyase (EC 4.2.2.13) | 36 |
| GH32 | invertase (EC 3.2.1.26); endo-inulinase (EC 3.2.1.7); β-2,6-fructan 6-levanbiohydrolase (EC 3.2.1.64); endo-levanase (EC 3.2.1.65); exo-inulinase (EC 3.2.1.80); fructan β-(2,1)-fructosidase/1-exohydrolase (EC 3.2.1.153); fructan β-(2,6)-fructosidase/6-exohydrolase (EC 3.2.1.154); | 23 |
| GH33 | sialidase or neuraminidase (EC 3.2.1.18); trans-sialidase (EC 2.4.1.-); anhydrosialidase (EC 4.2.2.15); Kdo hydrolase (EC 3.2.1.124); 2-keto-3-deoxynononic acid hydrolase / KDNase (EC 3.2.1.-) | 3 |
| GH35 | β-galactosidase (EC 3.2.1.23); exo-β-glucosaminidase (EC 3.2.1.165); exo-β-1,4-galactanase (EC 3.2.1.-); β-1,3-galactosidase (EC 3.2.1.-) | 22 |
| GH36 | α-galactosidase (EC 3.2.1.22); α-N-acetylgalactosaminidase (EC 3.2.1.49); stachyose synthase (EC 2.4.1.67); raffinose synthase (EC 2.4.1.82) | 13 |
| GH37 | [α,α-trehalase (EC 3.2.1.28).](http://www.enzyme-database.org/query.php?ec=3.2.1.28) | 7 |
| GH38 | α-mannosidase (EC 3.2.1.24); mannosyl-oligosaccharide α-1,2-mannosidase (EC 3.2.1.113); mannosyl-oligosaccharide α-1,3-1,6-mannosidase (EC 3.2.1.114); mannosyl-oligosaccharide α-1,3-mannosidase (EC 3.2.1.207); mannosyl-oligosaccharide α-1,6-mannosidase / exo-α-1,6-mannosidase (EC 3.2.1.163) | 4 |
| GH39 | α-L-iduronidase (EC 3.2.1.76); β-xylosidase (EC 3.2.1.37); α-L-arabinofuranosidase (EC 3.2.1.55); β-glucosidase (EC 3.2.1.21); β-galactosidase (EC 3.2.1.23); exo-β-1,4-glucanase / cellodextrinase (EC 3.2.1.74); 3-O-α-D-galactosyl-α-L-arabinofuranosidase (EC 3.2.1.215) | 7 |
| GH43 | β-xylosidase (EC 3.2.1.37); α-L-arabinofuranosidase (EC 3.2.1.55); xylanase (EC 3.2.1.8); α-1,2-L-arabinofuranosidase (EC 3.2.1.-); exo-α-1,5-L-arabinofuranosidase (EC 3.2.1.-); [inverting] exo-α-1,5-L-arabinanase (EC 3.2.1.-); | 97 |
| GH45 | endoglucanase (EC 3.2.1.4); xyloglucan-specific endo-β-1,4-glucanase / endo-xyloglucanase (EC 3.2.1.151); endo-β-1,4-mannanase (EC 3.2.1.78) | 4 |
| GH47 | [α-mannosidase (EC 3.2.1.113)](http://www.enzyme-database.org/query.php?ec=3.2.1.113) | 41 |
| GH49 | dextranase (EC 3.2.1.11); isopullulanase (EC 3.2.1.57); dextran 1,6-α-isomaltotriosidase (EC 3.2.1.95); sulfated arabinan endo-1,4-β-L-arabinanase (EC 3.2.1.-) | 1 |
| GH5 | endo-β-1,4-glucanase / cellulase (EC 3.2.1.4); endo-β-1,4-xylanase (EC 3.2.1.8); β-glucosidase (EC 3.2.1.21); β-mannosidase (EC 3.2.1.25); β-glucosylceramidase (EC 3.2.1.45) | 65 |
| GH51 | endoglucanase (EC 3.2.1.4); endo-β-1,4-xylanase (EC 3.2.1.8); β-xylosidase (EC 3.2.1.37); α-L-arabinofuranosidase (EC 3.2.1.55); cellobiohydrolase (EC 3.2.1.91) | 12 |
| GH53 | [endo-β-1,4-galactanase (EC 3.2.1.89).](http://www.enzyme-database.org/query.php?ec=3.2.1.89) | 3 |
| GH54 | α-L-arabinofuranosidase (EC 3.2.1.55); β-xylosidase (EC 3.2.1.37) | 2 |
| GH55 | exo-β-1,3-glucanase (EC 3.2.1.58); endo-β-1,3-glucanase (EC 3.2.1.39); laminarin-degrading enzyme (EC 3.2.1.-); hesperidin 6-O-α-L-rhamnosyl-β-glucosidase (EC 3.2.1.168) | 18 |
| GH6 | endoglucanase (EC 3.2.1.4); cellobiohydrolase (EC 3.2.1.91); lichenase / endo-β-1,3-1,4-glucanase (EC 3.2.1.73); | 2 |
| GH62 | [α-L-arabinofuranosidase (EC 3.2.1.55)](http://www.enzyme-database.org/query.php?ec=3.2.1.55) | 2 |
| GH63 | processing α-glucosidase (EC 3.2.1.106); α-1,3-glucosidase (EC 3.2.1.84); α-glucosidase (EC 3.2.1.20); mannosylglycerate α-mannosidase / mannosylglycerate hydrolase (EC 3.2.1.170); glucosylglycerate hydrolase (EC 3.2.1.208) | 2 |
| GH64 | [β-1,3-glucanase (EC 3.2.1.39)](http://www.enzyme-database.org/query.php?ec=3.2.1.39) | 12 |
| GH65 | α,α-trehalase (EC 3.2.1.28); maltose phosphorylase (EC 2.4.1.8); trehalose phosphorylase (EC 2.4.1.64); kojibiose phosphorylase (EC 2.4.1.230) | 3 |
| GH67 | α-glucuronidase (EC 3.2.1.139); xylan α-1,2-glucuronidase (EC 3.2.1.131) | 4 |
| GH7 | endo-β-1,4-glucanase (EC 3.2.1.4); reducing end-acting cellobiohydrolase (EC 3.2.1.176); chitosanase (EC 3.2.1.132); endo-β-1,3-1,4-glucanase (EC 3.2.1.73) | 17 |
| GH71 | [α-1,3-glucanase (EC 3.2.1.59)](http://www.enzyme-database.org/query.php?ec=3.2.1.59) | 20 |
| GH72 | [β-1,3-glucanosyltransglycosylase (EC 2.4.1.-)](http://www.enzyme-database.org/query.php?ec=2.4.1.*) | 11 |
| GH74 | endoglucanase (EC 3.2.1.4); oligoxyloglucan reducing end-specific cellobiohydrolase (EC 3.2.1.150); xyloglucanase (EC 3.2.1.151) | 6 |
| GH75 | [chitosanase (EC 3.2.1.132)](http://www.enzyme-database.org/query.php?ec=3.2.1.132) | 5 |
| GH76 | α-1,6-mannanase (EC 3.2.1.101); α-glucosidase (EC 3.2.1.20) | 27 |
| GH78 | α-L-rhamnosidase (EC 3.2.1.40); rhamnogalacturonan α-L-rhamnohydrolase (EC 3.2.1.174); L-Rhap-α-1,3-D-Apif -specific α-1,3-L-rhamnosidase (EC 3.2.1.-) | 67 |
| GH79 | β-glucuronidase (EC 3.2.1.31); hyaluronoglucuronidase (EC 3.2.1.36); heparanase (EC 3.2.1.166); baicalin β-glucuronidase (EC 3.2.1.167); β-4-O-methyl-glucuronidase (EC 3.2.1.-); glycyrrhizin β-glucuronidase (EC 3.2.1.128) | 8 |
| GH81 | [endo-β-1,3-glucanase (EC 3.2.1.39)](http://www.enzyme-database.org/query.php?ec=3.2.1.39) | 16 |
| GH88 | d-4,5-unsaturated β-glucuronyl hydrolase (EC 3.2.1.-); unsaturated chondroitin disaccharide hydrolase (EC 3.2.1.180); gellan tetrasaccharide unsaturated glucuronosyl hydrolase (EC 3.2.1.179) | 13 |
| GH93 | [exo-α-L-1,5-arabinanase (EC 3.2.1.-)](http://www.enzyme-database.org/query.php?ec=3.2.1.*) | 11 |
| GH94 | cellobiose phosphorylase (EC 2.4.1.20); laminaribiose phosphorylase (EC 2.4.1.31); cellodextrin phosphorylase (EC 2.4.1.49); chitobiose phosphorylase (EC 2.4.1.280); cellobionic acid phosphorylase (EC 2.4.1.321); β-1,2-oligoglucan phosphorylase (EC 2.4.1.333); 4-O-β-D-glucosyl-D-galactose phosphorylase (EC 2.4.1.-); solabiose phosphorylase (EC 2.4.1.389) | 2 |
| GH95 | α-L-fucosidase (EC 3.2.1.51); α-1,2-L-fucosidase (EC 3.2.1.63); α-L-galactosidase (EC 3.2.1.-) | 6 |
| **Total** | | **1301** |

**Table 6 (e): Annotation of putative genes identified in Carbohydrate-Active enzymes (CAZymes) in Glucosyl Transferases (GT)**

| **CAZyme family** | | **Annotation** | **Copy number** |
| --- | --- | --- | --- |
| **Glycosyl transferases (GT)** | | | |
| GT0 |  | | 26 |
| GT1 | UDP-glucuronosyltransferase (EC 2.4.1.17); zeatin O-β-xylosyltransferase (EC 2.4.2.40); 2-hydroxyacylsphingosine 1-β-galactosyltransferase (EC 2.4.1.45); N-acylsphingosine galactosyltransferase (EC 2.4.1.47) | | 77 |
| GT109 | [UDP-GalNAc: β-1,4-N-acetylgalactosaminyltransferase (EC 2.4.1.-)](http://www.enzyme-database.org/query.php?ec=2.4.1.*) | | 6 |
| GT15 | glycolipid 2-α-mannosyltransferase (EC 2.4.1.131); GDP-Man: α-1,2-mannosyltransferase (EC 2.4.1.-) | | 18 |
| GT17 | [β-1,4-mannosyl-glycoprotein β-1,4-N-acetylglucosaminyltransferase (EC 2.4.1.144)](http://www.enzyme-database.org/query.php?ec=2.4.1.144) | | 3 |
| GT2 | cellulose synthase (EC 2.4.1.12); chitin synthase (EC 2.4.1.16); dolichyl-phosphate β-D-mannosyltransferase (EC 2.4.1.83); dolichyl-phosphate β-glucosyltransferase (EC 2.4.1.117); | | 88 |
| GT20 | α,α-trehalose-phosphate synthase [UDP-forming] (EC 2.4.1.15); Glucosylglycerol-phosphate synthase (EC 2.4.1.213); trehalose-6-P phosphatase (EC 3.1.3.12); [retaining] GDP-valeniol: validamine 7-phosphate valeniolyltransferase (EC 2.-.-.-) | | 12 |
| GT21 | [UDP-Glc: ceramide β-glucosyltransferase (EC 2.4.1.80).](http://www.enzyme-database.org/query.php?ec=2.4.1.80) | | 2 |
| GT22 | Dol-P-Man: Man6GlcNAc2-PP-Dol α-1,2-mannosyltransferase (EC 2.4.1.259); Dol-P-Man: Man8GlcNAc2-PP-Dol α-1,2-mannosyltransferase (EC 2.4.1.261); Dol-P-Man: Man2-GlcNAc-phosphatidylinositol α-1,2-mannosyltransferase (EC 2.4.1.-); Dol-P-Man: Man3-GlcNAc-phosphatidylinositol α-1,2-mannosyltransferase (EC 2.4.1.-) | | 19 |
| GT24 | [UDP-Glc: glycoprotein α-glucosyltransferase (EC 2.4.1.-).](http://www.enzyme-database.org/query.php?ec=2.4.1.*) | | 6 |
| GT3 | [glycogen synthase (EC 2.4.1.11).](http://www.enzyme-database.org/query.php?ec=2.4.1.11) | | 6 |
| GT31 | N-acetyllactosaminide β-1,3-N-acetylglucosaminyltransferase (EC 2.4.1.149); Glycoprotein-N-acetylgalactosamine 3-β-galactosyltransferase (EC 2.4.1.122) | | 25 |
| GT32 | α-1,6-mannosyltransferase (EC 2.4.1.-); α-1,4-N-acetylglucosaminyltransferase (EC 2.4.1.-); α-1,4-N-acetylgalactosaminyltransferase (EC 2.4.1.-) | | 17 |
| GT33 | [GDP-Man: chitobiosyldiphosphodolichol β-mannosyltransferase (EC 2.4.1.142)](http://www.enzyme-database.org/query.php?ec=2.4.1.142) | | 4 |
| GT34 | UDP-Gal: galactomannan α-1,6-galactosyltransferase (EC 2.4.1.-); UDP-Xyl: xyloglucan α-1,6-xylosyltransferase (EC 2.4.2.39); α-1,2-galactosyltransferase (EC 2.4.1.-) | | 9 |
| GT35 | [glycogen or starch phosphorylase (EC 2.4.1.1).](http://www.enzyme-database.org/query.php?ec=2.4.1.1) | | 2 |
| GT39 | [Dol-P-Man: protein α-mannosyltransferase (EC 2.4.1.109)](http://www.enzyme-database.org/query.php?ec=2.4.1.109) | | 16 |
| GT4 | sucrose synthase (EC 2.4.1.13); sucrose-phosphate synthase (EC 2.4.1.14); α-glucosyltransferase (EC 2.4.1.52); lipopolysaccharide N-acetylglucosaminyltransferase (EC 2.4.1.56) | | 42 |
| GT41 | UDP-GlcNAc: peptide β-N-acetylglucosaminyltransferase (EC 2.4.1.255); UDP-Glc: peptide N-β-glucosyltransferase (EC 2.4.1.-); GDP-L-Fuc: protein O-α-L-fucosyltransferase (EC 2.4.1.221) | | 7 |
| GT48 | [1,3-β-glucan synthase (EC 2.4.1.34)](http://www.enzyme-database.org/query.php?ec=2.4.1.34) | | 9 |
| GT50 | [Dol-P-Man α-1,4-mannosyltransferase (EC 2.4.1.-)](http://www.enzyme-database.org/query.php?ec=2.4.1.*) | | 2 |
| GT51 | [murein polymerase (EC 2.4.1.129)](http://www.enzyme-database.org/query.php?ec=2.4.1.129) | | 2 |
| GT57 | [Dol-P-Glc: α-1,3-glucosyltransferase (EC 2.4.1.-)](http://www.enzyme-database.org/query.php?ec=2.4.1.*) | | 4 |
| GT58 | [Dol-P-Man: Man5GlcNAc2-PP-Dol α-1,3-mannosyltransferase (EC 2.4.1.258)](http://www.enzyme-database.org/query.php?ec=2.4.1.258) | | 9 |
| GT59 | [Dol-P-Glc: Glc2Man9GlcNAc2-PP-Dol α-1,2-glucosyltransferase (EC 2.4.1.256)](http://www.enzyme-database.org/query.php?ec=2.4.1.256) | | 3 |
| GT62 | α-1,2-mannosyltransferase (EC 2.4.1.-); α-1,6-mannosyltransferase (EC 2.4.1.-) | | 9 |
| GT64 | [UDP-GlcNAc: heparan α-N-acetylhexosaminyltransferase (EC 2.4.1.224)](http://www.enzyme-database.org/query.php?ec=2.4.1.224) | | 11 |
| GT66 | dolichyl-diphosphooligosaccharide - protein glycotransferase (EC 2.4.99.18); undecaprenyl-diphosphooligosaccharide - protein glycotransferase (EC 2.4.99.19) | | 3 |
| GT69 | [GDP-Man: α-1,3-mannosyltransferase (EC 2.4.1.-)](http://www.enzyme-database.org/query.php?ec=2.4.1.*) | | 17 |
| GT71 | [α-mannosyltransferase (EC 2.4.1.-)](http://www.enzyme-database.org/query.php?ec=2.4.1.*) | | 12 |
| GT76 | [Dol-P-Man: α-1,6-mannosyltransferase (EC 2.4.1.-)](http://www.enzyme-database.org/query.php?ec=2.4.1.*) | | 4 |
| GT8 | lipopolysaccharide α-1,3-galactosyltransferase (EC 2.4.1.44); UDP-Glc: (glucosyl)lipopolysaccharide α-1,2-glucosyltransferase (EC 2.4.1.-); lipopolysaccharide glucosyltransferase 1 (EC 2.4.1.58) | | 22 |
| GT84 | [cyclic β-1,2-glucan synthase (EC 2.4.1.-)](http://www.enzyme-database.org/query.php?ec=2.4.1.*) | | 2 |
| GT90 | UDP-Xyl: (mannosyl) glucuronoxylomannan/galactoxylomannan β-1,2-xylosyltransferase (EC 2.4.2.-); UDP-Glc: protein O-β-glucosyltransferase (EC 2.4.1.-); UDP-Xyl: protein O-β-xylosyltransferase (EC 2.4.2.-) | | 22 |
| GT95 | [UDP-β-L-Araf:hydroxyproline β-L-arabinofuranosyltransferase (EC 2.4.2.-)](http://www.enzyme-database.org/query.php?ec=2.4.2.*) | | 2 |
| **Total** | | | **518** |

**Table 6 (f): Annotation of putative genes identified in Carbohydrate-Active enzymes (CAZymes) in Polysaccharide Lyases (PL)**

| **CAZyme family** | | **Annotation** | **Copy number** |
| --- | --- | --- | --- |
| **Polysaccharide lyases (PL)** | | | |
| PL1 | pectate lyase (EC 4.2.2.2); exo-pectate lyase (EC 4.2.2.9); pectin lyase (EC 4.2.2.10) | | 32 |
| PL11 | rhamnogalacturonan endolyase (EC 4.2.2.23); rhamnogalacturonan exolyase (EC 4.2.2.24) | | 1 |
| PL15 | alginate lyase (EC 4.2.2.3); oligoalginate lyase / exo-alginate lyase (EC 4.2.2.26); heparin lyase / heparin lyase I (EC 4.2.2.7); heparin-sulfate lyase / heparin lyase III (EC 4.2.2.8) | | 1 |
| PL20 | [endo-β-1,4-glucuronan lyase (EC 4.2.2.14)](http://www.enzyme-database.org/query.php?ec=4.2.2.14) | | 1 |
| PL26 | [rhamnogalacturonan exolyase (EC 4.2.2.24)](http://www.enzyme-database.org/query.php?ec=4.2.2.24) | | 6 |
| PL3 | [pectate lyase (EC 4.2.2.2)](http://www.enzyme-database.org/query.php?ec=4.2.2.2) | | 15 |
| PL4 | [rhamnogalacturonan endolyase (EC 4.2.2.23)](http://www.enzyme-database.org/query.php?ec=4.2.2.23) | | 14 |
| PL42 | L-rhamnose-α-1,4-D-glucuronate lyase (EC 4.2.2.-); L-Rhα-α-1,4-GlcA α-L-rhamnohydrolase (EC 3.2.1.-) | | 6 |
| PL7 | poly(β-mannuronate) lyase / M-specific alginate lyase (EC 4.2.2.3); α-L-guluronate lyase / G-specific alginate lyase (EC 4.2.2.11); poly-(MG)-lyase / MG-specific alginate lyase (EC 4.2.2.-); endo-β-1,4-glucuronan lyase (EC 4.2.2.14); oligoalginate lyase / exo-alginate lyase (EC 4.2.2.26) | | 1 |
| PL9 | pectate lyase (EC 4.2.2.2); exopolygalacturonate lyase (EC 4.2.2.9); thiopeptidoglycan lyase (EC 4.2.2.-); rhamnogalacturonan endolyase (EC 4.2.2.23) | | 5 |
| **Total** | | | **82** |
